# Preclinical Efficacy and Safety Study of a Novel Dermal Fibroblast Modulating Drug, SLI‐F06, in Cutaneous Wound Healing

**DOI:** 10.1002/mco2.70761

**Published:** 2026-05-24

**Authors:** Zhong Zheng, Pin Ha, Chenshuang Li, Grace Xinlian Chang, Wenlu Jiang, Xiaoxiao Pang, Zhaohan Zeng, Elisabeth Leeflang, Kang Ting, Chia Soo

**Affiliations:** ^1^ Scarless Laboratories, Inc. Torrance California USA; ^2^ Department of Periodontics, School of Dental Medicine University of Pennsylvania Philadelphia Pennsylvania USA; ^3^ Division of Plastic and Reconstructive Surgery, Department of Surgery, David Geffen School of Medicine University of California Los Angeles California USA; ^4^ Department of Orthopaedic Surgery and the Orthopaedic Hospital Research Center, David Geffen School of Medicine University of California Los Angeles California USA; ^5^ Department of Orthodontics, School of Dental Medicine University of Pennsylvania Philadelphia Pennsylvania USA; ^6^ Department of Surgery, David Geffen School of Medicine University of California Los Angeles California USA; ^7^ Chongqing Key Laboratory of Oral Diseases and Biomedical Sciences, Chongqing Municipal Key Laboratory of Oral, Biomedical Engineering of Higher Education Stomatological Hospital of Chongqing Medical University Chongqing China; ^8^ American Dental Association Forsyth Institute Cambridge Massachusetts USA; ^9^ Department of Bioengineering, Henry Samueli School of Engineering and Applied Science University of California Los Angeles California USA

**Keywords:** fibroblasts, fibromodulin (FMOD), SLI‐F06, transforming growth factor β (TGFβ), wound healing

## Abstract

Scarring results in significant developmental, functional, aesthetic, and psychological challenges. Despite substantial demand from patients and healthcare providers, no drugs or biologics are currently approved specifically for preventing or reducing scarring. Our previous studies indicate that fibromodulin (FMOD) modulates adult dermal fibroblasts to adopt fetal‐like characteristics, thereby improving wound appearance, reducing scar size, and enhancing tensile strength in adult skin healing. To address the high costs, variability, and safety concerns of producing FMOD through mammalian cells, a novel, chemically synthesized FMOD‐derived peptide, SLI‐F06, has been developed. SLI‐F06 retains FMOD's essential properties, such as promoting cell migration, increasing tensile strength, and stimulating antifibrotic effects. Comprehensive animal studies using models such as mice, rats, Yorkshire pigs (the standard for normal human wound healing), and red Duroc pigs (closely mimicking human proliferative and hypertrophic scarring) demonstrate significant improvements in scar appearance, tensile strength tests, and histological outcomes with SLI‐F06. Additionally, a formulation buffer has been developed to maintain physiological pH and osmolality, ensuring the stability of SLI‐F06 for a suitable duration in clinical settings after removal from refrigeration. SLI‐F06 exhibits no genotoxicity or local or systemic toxicity in extensive studies required by the United States Food and Drug Administration for Investigational New Drug applications.

## Graphical abstract

A novel, chemically synthesized fibromodulin (FMOD)‐derived peptide, SLI‐F06, has been developed to significantly improve scar appearance, tensile strength, and histological outcomes among multiple preclinical animal models. Demonstrating no genotoxicity or systemic/local toxicity, SLI‐F06 has received United States Food and Drug Administration approval as an Investigational New Drug (IND).

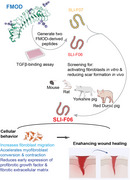

## 1 Introduction

Each year, cutaneous fibrosis (scarring) affects approximately 100 million patients in the United States [1], with annual management costs exceeding $20 billion [2]. If not properly addressed, scarring can lead to significant developmental, functional, aesthetic, and psychological challenges. Physically, excessive scarring may cause pain, itching, discomfort, and contractures that limit mobility and daily activities [3]. Severe hypertrophic scarring (HS) is even able to result in skeletal erosion and lifelong disability [4]. Furthermore, patients with disfiguring conditions often experience anxiety, social avoidance, and a decreased quality of life, impacting their psychological well‐being [5]. Robust evidence indicates that scarring can lead to interpersonal rejection, negatively affecting intimate relationships [6]. Therefore, there is an enormous demand for safe and effective scar reduction treatments from patients, caregivers, and wound repair experts. A recent survey of over 24,000 physicians who routinely perform cosmetic and reconstructive plastic surgery procedures revealed that 91% of postoperative patients were dissatisfied with their scars and desired even minor improvement [3]. Unfortunately, current scar prevention and reduction strategies have minimal effectiveness or undesirable side effects [7].

In an effort to develop better strategies for excessive scarring, we explored potential solutions from developmental biology, explicitly drawing insights from fetal scar repair mechanisms known for their scarless healing. Through comprehensive animal models, including mice, rats, and pigs, we identified that fibromodulin (FMOD), an extracellular matrix small leucine‐rich proteoglycan, is critical for scarless fetal wound healing [8]. FMOD promotes key regenerative processes in skin wound healing, leading to improved gross appearance, reduced scarring, and accelerated recovery of tensile strength and myofibroblast clearance [9, 10, 11]. However, producing FMOD in bacteria and yeast cultures is inefficient, necessitating mammalian cell culture production [12] with attendant drawbacks of higher costs, lot variability, and safety concerns (e.g., the presence of adventitious agents associated with mammalian cell production).

Notably, the National Institutes of Health (NIH) recognizes that peptide therapies offer several inherent advantages over their respective protein counterparts, including higher specificity and selectivity, lower toxicity, immunogenicity, and tissue accumulation, and weaker drug–drug interactions [13, 14, 15]. Meanwhile, peptides can be efficiently synthesized using various strategies, allowing for controlled and inexpensive production compared with recombinant proteins. This is especially relevant to FMOD protein, which contains many disulfide bonds and posttranslational modifications [16], making it challenging to synthesize and purify commercially.

Meanwhile, FMOD is known to directly bind to transforming growth factor (TGF)βs [12], and our previous studies have shown that fine‐tuning different TGFβ1‐responsive genes to elicit a more “fetal‐like” phenotype is important for FMOD to exert its antiscarring biopotency in adult wound models [9]. At the molecular level, FMOD reduces TGFβ1 expression while accelerating connective tissue growth factor (CTGF) and α‐smooth muscle actin (α‐SMA) expression. At the cellular level, FMOD increases fibroblast migration and myofibroblast differentiation and contraction [9]. Given these findings, FMOD derivatives that bind to TGFβ1 were considered potential bioactive peptides.

In this study, we first identified the potential TGF‐β1‐binding regions of FMOD and used this information to develop proprietary, chemically synthesized FMOD‐derived peptides, designated SLI‐F06 and SLI‐F07. We subsequently confirmed that SLI‐F06 retains promigration, protensile strength, and antifibrotic properties comparable to the full‐length FMOD protein [9]. More importantly, the antiscarring efficacy of FMOD was successfully recapitulated by SLI‐F06 in multiple rodent and porcine preclinical wound models, across different formulation buffers, with a favorable safety profile. This product, therefore, combines the advantages of peptide therapeutics with the established potential of FMOD, offering a promising approach for the management of excessive scarring.

## 2 Results

### 2.1 Two Synthesized FMOD‐Derived Peptides Were Identified Based on Their TGFβ‐Binding Activities

Producing FMOD in bacterial systems is challenging [12], so we employed the small ubiquitin‐like modifier (SUMO)‐fusion system [17] to generate soluble FMOD fragments in *Escherichia coli* for the initial TGFβ1‐binding screening. Among the putative regions of human FMOD that bind to TGFβ1, regions E and F exhibited high TGFβ1‐binding potency (Figure S1). Two peptides derived from these regions, SLI‐F06 and SLI‐F07, were chemically synthesized. Since dimethyl sulfoxide (DMSO) is an important polar aprotic solvent capable of dissolving both polar and nonpolar compounds, it is particularly effective for dissolving peptides with polar and nonpolar parts [18]. Thus, these two chemically synthesized peptides were dissolved in 5% DMSO and adjusted to the final concentration in phosphate‐buffered saline (PBS) for the subsequent in vitro tests and the initial animal efficacy investigations described in the following sections.

In the subsequent screening for binding all three mammalian TGFβ isoforms, SLI‐F06 and SLI‐F07 exhibited differential binding activities (Figure S2). Notably, SLI‐F07 demonstrated binding to TGFβ1 at the 1:1 ratio (mol:mol), under which neither SLI‐F06 nor the whole FMOD protein showed binding activity (Figure S2A). SLI‐F06 did not demonstrate the capability to bind to TGFβ2 (Figure S2B). In comparison, SLI‐F07 displayed superior TGFβ3‐binding potency compared with SLI‐F06 and FMOD (Figure S2C). These findings suggest potentially different biopotencies of these synthesized FMOD‐derived peptides.

### 2.2 The FMOD‐Derived Peptide SLI‐F06 Maintained the Promigratory and Procontractile Effects of FMOD on Adult Rat Dermal Fibroblasts

Similar to FMOD [9], SLI‐F06 and SLI‐F07 had minimal impact on rat dermal fibroblast (RDF) proliferation up to a concentration of 200 nM when administered alone (Figure S3). However, SLI‐F06 promoted RDF proliferation at higher concentrations, whereas SLI‐F07 and FMOD [9] reduced it. In the presence of TGFβ1, SLI‐F06 stimulated RDF proliferation (Figure S3A), mirroring the effect of FMOD in the same context [9]. In contrast, SLI‐F07 intensified the inhibitory effects of TGFβ1 on RDFs (Figure S3B).

**FIGURE 1:**
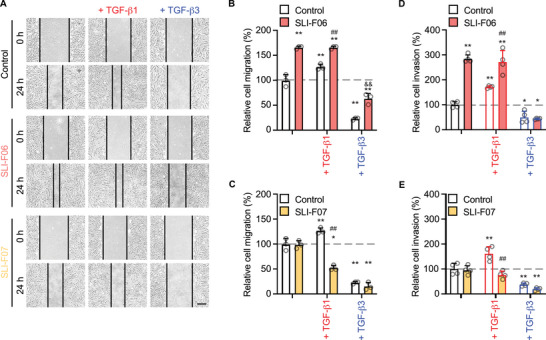
Different effects of synthesized FMOD‐derived peptides on adult RDF migration and invasion. Before and 24 h after pipette‐tip scratching, photos were taken to document the cell migration (A) under the influence of synthesized FMOD‐derived peptide SLI‐F06 (B) or SLI‐F07 (C) with or without the coapplication of TGFβ1 or TGFβ3. Meanwhile, a transwell assay assessed the effects of SLI‐F06 (D) and SLI‐F07 (E) on RDF invasion. Scale bar = 400 µm. Data were normalized to untreated RDFs (dashed lines). *N* = 3 (B and C) or 4 (D and E); *, *p* < 0.05; **, *p* < 0.005, respectively, compared with the vehicle buffer control; ^##^, *p* < 0.005, compared with the group treated with TGFβ1 alone; and ^&&^, *p* < 0.005, compared with the group treated with TGFβ3 alone.

To eliminate the potential influence of cell proliferation, we used 200 nM SLI‐F06 or SLI‐F07 to assess their effects on adult RDF migration and invasion (Figure 1). Akin to FMOD [9, 19], SLI‐F06 significantly enhanced RDF migration in the presence of TGFβ1 and partially counteracted the inhibitory effects of TGFβ3 (Figure 1A,B). Notably, SLI‐F06 alone stimulated RDF migration to a level comparable to that observed with additional TGFβ1 application, further highlighting its promigratory potency. In contrast, SLI‐F07 did not promote RDF migration or reverse the TGFβ3‐induced inhibition (Figure 1A,C). SLI‐F07 also negated the elevated migration caused by TGFβ1. Similar effects were noticed in the invasion assay (Figure 1D,E), although 200 nM SLI‐F06 could not alleviate TGFβ3's inhibition on adult RDF invasion.

**FIGURE 2:**
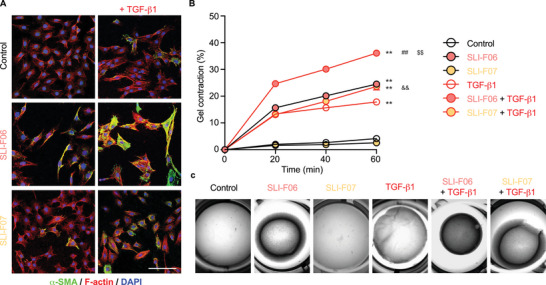
Different effects of synthesized FMOD‐derived peptides on adult RDF‐derived myofibroblast conversion and contraction. After 48 h treatment, α‐SMA staining was used to assess the conversion of RDF to myofibroblasts. F‐actin was used to elucidate the cell boundary and DAPI for the nuclear counterstaining (A). Myofibroblast contraction in 3D collagen gel was documented for 60 min (B), and the representative photos at the end timepoint were shown (C). Scale bar = 100 µm. *N* = 6; **, *p* < 0.005, compared with the solution vehicle control; ^##^, *p* < 0.005, compared with the group treated with SLI‐F06 alone; ^&&^, *p* < 0.005, compared with the group treated with SLI‐F07 alone; and ^$$^, *p* < 0.005, compared with the group treated with TGFβ1 alone.

In line with FMOD [9], both SLI‐F06 and SLI‐F07 significantly enhanced the conversion of TGFβ1‐stimulated RDF to the myofibroblasts, as evidenced by α‐SMA staining (Figure 2A), that normally function to generate contractile forces to reduce the wound defect size [20]. Unlike FMOD, SLI‐F06 or SLI‐F07 alone could promote RDF‐myofibroblast conversion without additional TGFβ1. However, more intense α‐SMA staining and more apparent stress fiber formation were observed in myofibroblasts treated with SLI‐F06 and TGFβ1 together, indicating a more contractile phenotype (Figure 2A). Supporting the staining results, quantitative 3D collagen gel contraction assays confirmed that SLI‐F06 significantly potentiates myofibroblast contractility, independent of TGFβ1 coapplication (Figure 2B,C). On the other hand, the effect of SLI‐F07 on myofibroblast contractility was negligible.

Next, TGFβ1‐related gene expression was employed for further screening validation. SLI‐F06 demonstrated a similar inhibitory effect as FMOD [9] on the auto‐induction of TGFβ1 in adult RDFs at early time points (2 and 6 h posttreatment). Concurrently, the combined treatment of SLI‐F06 and TGFβ1 resulted in an upregulation of *Tgfβ1* at later time points (24 and 48 h posttreatment), occurring after the TGFβ1‐induced *Tgfβ1* expression had returned to the baseline levels (Figure S4A). Additionally, akin to FMOD [9], SLI‐F06 amplified and extended the expression of a subset of TGFβ1‐downstream genes crucial for fibroblast migration/invasion and myofibroblast conversion and contraction, including *Ctgf* (encoding CTGF), *Acta2* (encoding α‐SMA), and *Mmp2* (encoding matrix metalloproteinase 2), even in the absence of exogenous TGFβ1 (Figure S4B–D). In contrast, SLI‐F07 alone elevated *Tgfβ1* expression and maintained a relatively stable *Tgfβ1* level in RDFs, regardless of TGFβ1 coapplication (Figure S4A). Unlike FMOD and SLI‐F06, SLI‐F07 attenuated the *Ctgf* and *Acta2* elevation in response to TGFβ1 stimulation (Figure S4B,C) and did not influence *Mmp2* expression (Figure S4D). Therefore, while SLI‐F06 and FMOD selectively orchestrated the TGFβ1 downstream pathway, SLI‐F07 appeared to buffer the TGFβ1 signal transduction. It is also noteworthy that SLI‐F06 stimulated endogenous *Fmod* expression reduced by exogenous TGFβ1 application (Figure S4E), whereas the effect of SLI‐F07 was considerably less pronounced.

These findings underscore the distinct roles of SLI‐F06 and SLI‐F07 in modulating adult dermal fibroblast dynamics and their potential roles in wound healing.

### 2.3 SLI‐F06 Successfully Emulated the Advantages of FMOD Across Various Established Adult Animal Wound Models

To assess the in vivo efficacy of SLI‐F06 and SLI‐F07 for the prevention of excessive scarring, we utilized a variety of established adult animal wound models [9, 19, 21, 22, 23]. The application of 2 mg/mL SLI‐F06 not only significantly reduced the scar size (assessed by previously established scar index [9, 10, 19]; Figure S5) in adult wildtype mice but also partially alleviated excessive scar formation in *Fmod^−/−^* mice (Figure S6). This scar reduction effect of SLI‐F06 was consistently observed in both adult rat model (Figure S7) and adult Yorkshire pig model (Figure S8), which closely represents normal human cutaneous wound healing. These findings from multiple animal model efficacy studies provide a promising starting point for developing SLI‐F06 as a therapeutic to prevent excessive scarring. In contrast, SLI‐F07 did not demonstrate a noticeable antiscar effect across these models. Consequently, we infer that SLI‐F07 may not be efficacious in reducing scar formation.

**FIGURE 3:**
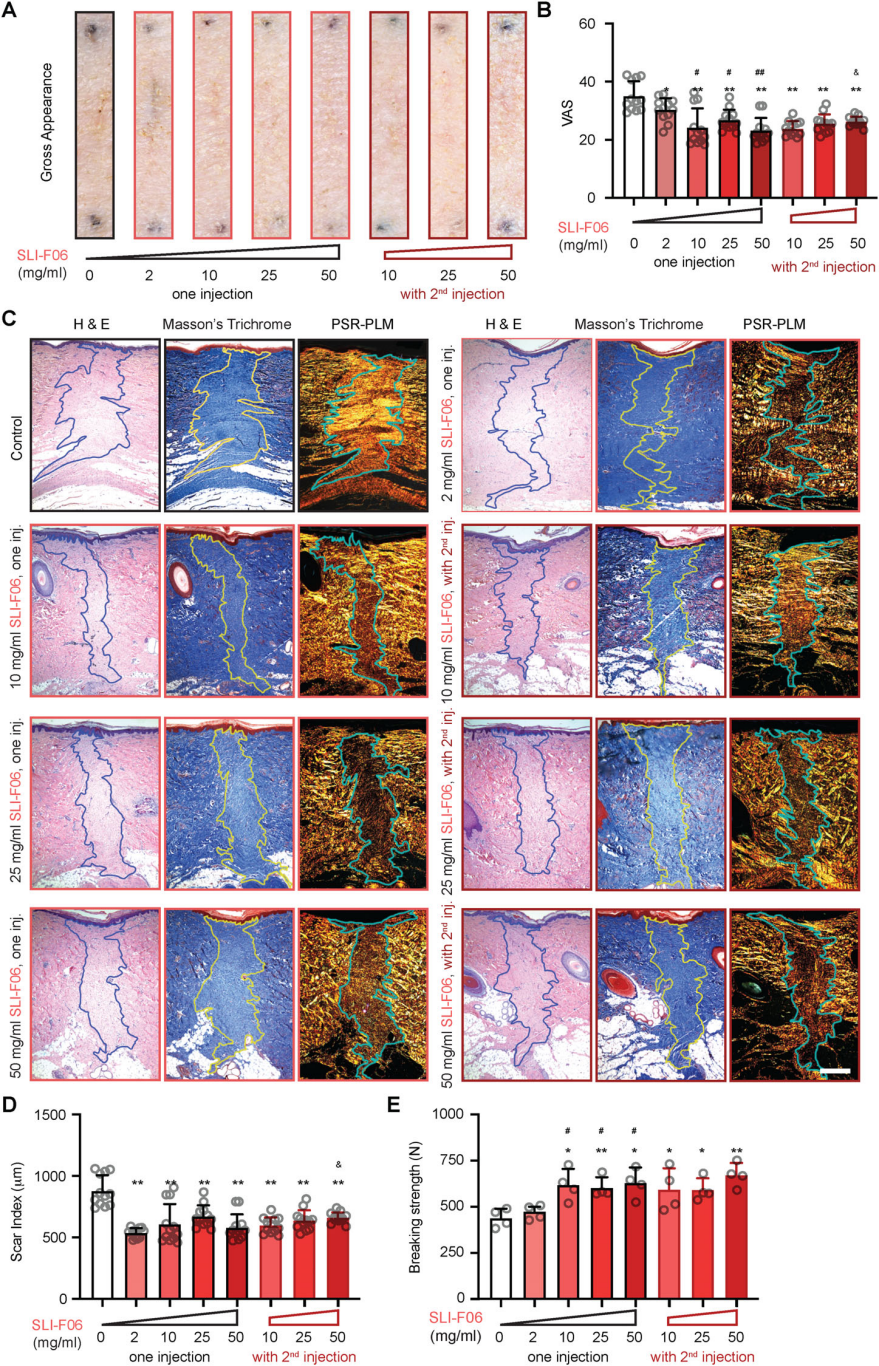
Optimization of SLI‐F06 dose and regimen in normal‐mechanical‐loading wound model of adult Yorkshire pigs. Normal‐mechanical‐loading (0.5 cm‐width × 1.5 cm‐length) primarily closed wounds were created on adult female Yorkshire pig dorsal skin. 100 µL of SLI‐F06 was intradermally injected into each wound edge at Time 0 only for one injection group and another 50 µL 24 h later for the two‐injection groups. One injection of vehicle buffer was used as control. Representative scar appearance was documented at 8 weeks postinjury (A), and isual Analogue Score (VAS) was determined by three Doctor of Medicine (MD) reviewers (B). Hematoxylin and eosin (H&E) staining, Masson's Trichrome staining, and PSR–PLM images were taken at 8 weeks (C) to quantify the scar size (D). Blue (on H&E staining), yellow (on Masson's Trichrome staining), and cyan [on Picrosirius red (PSR)–polarized light microscopy (PLM) image] lines outline scars. Breaking strength was also measured to represent the wound tensile strength (E). Scale bar = 500 µm. *N* = 12 (B and D) or 4 (E); *, *p *< 0.05, **, *p* < 0.005, compared with the vehicle buffer control; ^#^, *p* < 0.05, ^##^, *p* < 0.005, compared with the group treated with one‐injection of 2 mg/mL SLI‐F06; and ^&^, *p* < 0.05, compared with the group treated with one‐injection of the same concentration of SLI‐F06.

Subsequently, we increased the concentration of SLI‐F06 to determine its effective dosage in the 0.5 cm‐width normal Yorkshire pig primarily closed wounds [9]. We also administered a second injection at half the strength to evaluate whether repeated dosing could enhance the beneficial impact of SLI‐F06 on skin wound healing (Figure 3). As anticipated, administrating SLI‐F06 at all tested concentrations led to an improved gross visual appearance (Figure 3A,B) and a reduction in scar size (Figure 3C,D) compared with the vehicle buffer control. However, 2 mg/mL SLI‐F06 did not considerably benefit wound tensile strength while higher concentrations of SLI‐F06, ≥10 mg/mL, markedly increased the wound breaking strength relative to the control (Figure 3E). Notably, the scar reduction effects of SLI‐F06 were only marginally increased at concentrations of 10 mg/mL or higher, regardless of whether a second injection was applied (Figure 3). Therefore, a single injection of 10 mg/mL was considered the optimal dose and regimen for SLI‐F06, effectively improving gross visual appearance, reducing scar size, and accelerating wound tensile strength reestablishment.

### 2.4 The Formulation Buffer for SLI‐F06 Has Been Optimized to Minimize Risks Associated With Human Use

While DMSO is frequently employed to dissolve peptides due to its unique solvent properties, it is crucial to note that peptides containing cysteine and methionine are unstable in DMSO [18]. Moreover, numerous dermatological and allergic reactions to DMSO have been reported in humans [24], necessitating the development of a DMSO‐free formulation to mitigate risks associated with human usage. Meanwhile, citrate buffers were widely utilized in the pharmaceutical industry as they can be employed over a wide pH range (pH 3.0–7.4), do not crystallize during the lyophilization and remain amorphous with minimal pH changes, and are likely to protect the peptide drug by inhibiting proteolysis and preventing protein/peptide from precipitation—a common issue with many other types of buffers [25]. Therefore, following extensive research and optimization efforts, we have adopted the replacement formulation buffer composed of 0.1 M citrate–Na_2_HPO_4_, pH 7.4 with 0.9% (wt/vol) NaCl. In this formulation, NaCl is employed to establish a biocompatible osmotic environment, while a pH of 7.4 was selected to create the physiological pH. This formulation buffer facilitates the creation of a stable SLI‐F06 solution at concentrations reaching up to 25 mg/ml. When prepared in bulk (50 mL), the potency of SLI‐F06, as determined by high‐performance liquid chromatography (HPLC), remained consistent over a 24‐h period under refrigeration at 5°C (Figure S9). At 25°C/60% relative humidity, the normal storage condition recognized by the International Council for Harmonisation (ICH), the potency of SLI‐F06 was virtually unchanged in the first 8 h, while a minor decrease in potency to below 95% was observed after 24 h.

### 2.5 The Effectiveness of SLI‐F06 for Scar Reduction Was Confirmed in Primary Intention Wound Closure Models in HS‐Prone Red Duroc Pigs

**FIGURE 4:**
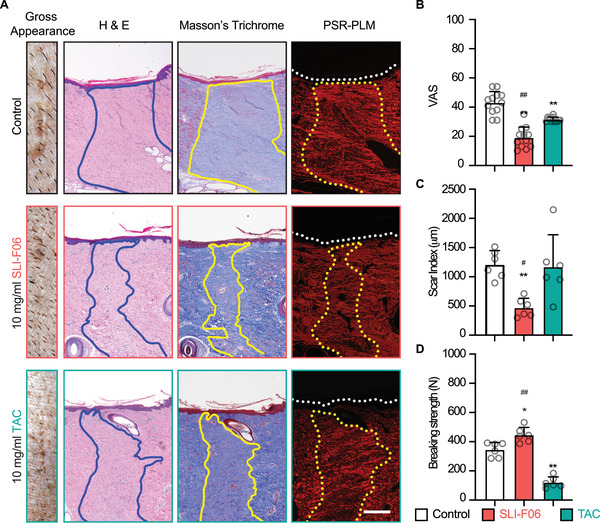
Efficacy of SLI‐F06 in formulation buffer assessed on normal‐mechanical‐loading, primary intention wounds of adult female red Duroc pigs at 8 weeks postinjury. The gross visual appearance of normal‐mechanical‐loading (0.5 cm‐width × 1.5 cm‐length) wounds with one injection of formulation buffer (control), 10 mg/mL SLI‐F06 in formulation buffer, or 10 mg/mL TAC is shown, along with the corresponding histological evaluation by H&E staining, Masson's trichrome staining, and PSR–PLM images. Blue (on H&E staining) and yellow (on Masson's Trichrome staining and PSR–PLM image) lines outline scars (A). The VAS (B) and scar index (C) quantified the gross visual appearance and scar size, respectively. Tensile strength was assessed as breaking strength (D). Scale bar = 0.5 mm. *N* = 12 (B), or 6 (C and D); *, *p* < 0.05; **, *p* < 0.005, compared with the formulation buffer control; and ^#^, *p* < 0.05, ^##^, *p* < 0.005, compared with the group treated with 10 mg/mL TAC.

The efficacy of SLI‐F06 in the refined formulation buffer was then validated in HS‐prone adult red Duroc pigs [10]. We also compared SLI‐F06 with triamcinolone acetonide (TAC), a corticosteroid broadly used to prevent and suppress scar formation [26]. SLI‐F06 and TAC improved the gross visual appearance of the 0.5 cm‐width primarily closed wounds of red Duroc pigs; however, SLI‐F06 achieved a more pronounced improvement (Figure 4A,B). While TAC failed to reduce the scar size (Figure 4C) and weakened the wound tensile strength by 65% (Figure 4D), SLI‐F06 significantly decreased the scar size and enhanced the wound tensile strength compared with the formulation buffer control and TAC (Figure 4).

**FIGURE 5:**
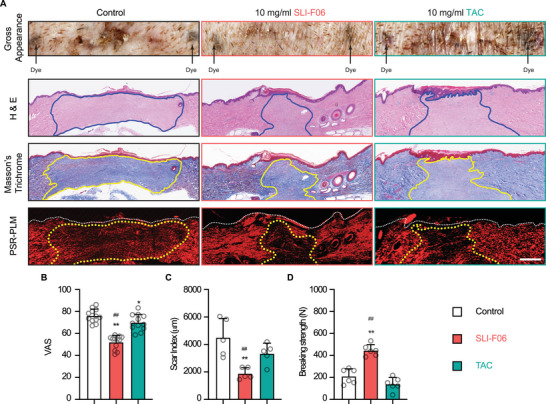
Efficacy of SLI‐F06 in formulation buffer confirmed on excessive‐mechanical‐loading, primary intention wounds of adult female red Duroc pigs at 8 weeks postinjury. The gross visual appearance of excessive‐mechanical‐loading (2.0 cm‐width × 1.5 cm‐length) wounds with one injection of formulation buffer (control), 10 mg/mL SLI‐F06 in formulation buffer, or 10 mg/mL TAC is shown, along with the corresponding histological evaluation by H&E staining, Masson's trichrome staining, and PSR–PLM images. Blue (on H&E staining) and yellow (on Masson's Trichrome staining and PSR–PLM image) lines outline scars (A). The VAS (B) and scar index (C) quantified the gross visual appearance and scar size, respectively. Tensile strength was assessed as breaking strength (D). Scale bar = 1.0 mm. *N* = 12 (B), 5 (C), or 6 (D); *, *p* < 0.05; **, *p* < 0.005, compared with the formulation buffer control; and ^##^, *p* < 0.005, compared with the group treated with 10 mg/mL TAC.

Furthermore, a growing body of evidence suggests that excessive mechanical loading across wounds significantly triggers HS formation [4, 27, 28]. To further validate the scar reduction potency of SLI‐F06, we utilized the established excessive‐mechanical‐loading (2.0 cm‐width × 1.5 cm‐length) wound model in red Duroc pigs [10]. In this model, TAC only marginally improved the gross visual appearance of the wound by 3% and reduced the scar size by 33% while concurrently decreasing the wound tensile strength by 27% (Figure 5). In striking contrast, SLI‐F06 not only significantly improved the gross visual appearance of the wound by 35%, but also markedly reduced the scar size by 56% compared with the formulation buffer control (Figure 5A–C). SLI‐F06 also resulted in a substantial 160% increase in tensile strength in excessive mechanical loading wounds of red Duroc pigs (Figure 5D). These findings underscore the potential of SLI‐F06 as a promising therapeutic agent in scar management, particularly when rapid establishment of wound tensile strength is preferred.

### 2.6 Preclinical Safety Tests Demonstrated the Safety of SLI‐F06 for Further Clinical Studies in Humans

Next, we conducted preclinical safety studies in collaboration with the experienced contract research organizations (CRO)s, Toxikon Corporation (Bedford, MA) and Charles River Laboratories (Ashland, OH), with the good laboratory practice (GLP)‐compliance (Table 1). These studies include 5‐ and 28‐day intravenous bolus repeat‐dosing in rats [up to a maximum feasible dose (MFD) of 125 mg/kg/day], a 5‐day subcutaneous repeat‐dosing in pigs (up to an MFD of 125 mg/kg/day), and a 3‐day intradermal repeat‐dosing in a wounded pig model (up to an MFD of 60 mg/kg/day). All toxicology studies demonstrated no observable SLI‐F06‐related adverse effects, even at the dose of MFDs.

**TABLE 1:** Preclinical safety tests of SLI‐F06.^a^

Study type	Route of administration	Concentration/dose	Study results
28‐day repeated dose in rats with 14‐day recovery (Table S1)	Intravenous	0, 50, 90, or 125^b^ mg/kg/day	No adverse effects in functional observational battery (FOB); no‐observed‐adverse‐effect level (NOAEL) > 125 mg/kg/day
5‐day repeated subcutaneous dose in Göttengen minipigs with 7‐day recovery (Table S2)	Subcutaneous	0, 10, 78, or 125^b^ mg/kg/day	No adverse effects in FOB; no electrocardiogram (EGC) findings; NOAEL > 125 mg/kg/day
3‐day repeated intradermal dose in wounded Göttengen minipigs (Table S3)	Intradermal	0, 24, or 60^b^ mg/kg/day	No adverse effects in FOB; NOAEL > 60 mg/kg/day
Pulmonary safety pharmacology in conscious rats (Table S4)	Intravenous	0, 50, 90, or 125^b^ mg/kg/day	No significant effects on the pulmonary system
Genotoxicity: Ames assay (Table S5)	In vitro	0.0024, 0.0076, 0.0228, 0.068, 0.205, 0.617, 1.851, 5.555, 16.666, and 50 mg/mL	No safety signals for potential carcinogenicity
Local tolerance: Episkin test	In vitro	25 mg in 25 µL Dulbecco's PBS (1 mg/µL)	No observed negative local effects

^a^ All tests are GLP compliant. Comprehensive details of these studies are documented in Supporting Information.

^b^ Maximum feasible dose (MFD).

It is well known that toxicokinetic (TK) data are critical in preclinical safety assessments, as they provide essential exposure context for interpreting toxicity, selecting safe doses, and meeting regulatory expectations, thereby protecting clinical trial participants. Therefore, TK studies were also conducted in SLI‐F06's preclinical safety assessment. Rat TK analysis revealed that no quantifiable levels of SLI‐F06 were detectable 8 h postdose at MFD, and this detection time was further reduced to 4 h at lower tested doses. A slight accumulation was only observed in the 125 mg/kg MFD dose group via bolus intravenous injection, as the increased half‐life values suggested (Table S6). Pig studies showed a dose‐proportional increase in the maximum observed concentration (*C*_max_) (Tables S7 and S8). In addition, the pig TK data indicated that the half‐life (*t*_1/2_) values of SLI‐F06 administered via the subcutaneous route were less than 5 h, while the no‐observed‐adverse‐effect level was at least 125 mg/kg/day (MFD; Table S7). Notably, *C*_max_ did not increase from Day 1 to Day 5, indicating no systemic accumulation of SLI‐F06 from Day 1 to Day 5 in pigs that received a subcutaneous injection of SLI‐F06 (Table S7). When SLI‐F06 was administered intradermally in wounded pigs, no plasma SLI‐F06 was detected after 4 h postdose, while the average half‐life was shorter than 2 h (Table S8).

Additionally, the *Salmonella typhimurium* and *E. coli* reverse mutation assay was performed to evaluate the genotoxicity of SLI‐F06 (Table S5). The results demonstrated that exposure to SLI‐F06 was not associated with any toxicity and did not increase the frequency of revertant at any test concentrations in any of the strains tested, regardless of the presence or absence of metabolic activation (Table 1). Meanwhile, Episkin testing revealed no local irritation induced by SLI‐F06 (Tables 1 and S9). Furthermore, a pulmonary safety assessment conducted in conscious rats demonstrated that intravenous administration of SLI‐F06 at 50 mg/kg produced no biologically relevant effects on respiratory rate, tidal volume, or minute volume (Table S10). At the higher dose of 90 mg/kg, a slight, transient decrease in minute volume was noted. Although the MFD (125 mg/kg) also resulted in a modest reduction in minute volume compared with the formulation buffer control, no SLI‐F06‐related effects on respiratory rate or tidal volume were observed at this dose. Thus, the CRO conducting the study concluded that SLI‑F06 had no significant effects on the pulmonary system (Table 1).

### 2.7 Preliminary Chemistry, Manufacturing, and Controls Activities Have Been Conducted to Support the Investigational New Drug (IND) Application for SLI‐F06

Chemistry, manufacturing, and controls (CMC) data are a critical component of an investigational new drug (IND) application submitted to the United States Food and Drug Administration (US FDA). With the support of the CRO, PolyPeptide Group, current GMP‐grade SLI‐F06 synthesis has been optimized, achieving the US FDA's purity requirement for pharmacological usage [29, 30]. An analytical method for SLI‐F06, covering a concentration range of 25–1000 µg/mL, has been developed and validated using reverse‐phase HPLC with ultraviolet detection, in compliance with GLP regulations. Stability testing conducted using this method demonstrated minimal degradation of SLI‐F06, thereby meeting the requirements outlined in the relevant ICH guidance [31] and satisfying the acceptability criteria of the US FDA [29]. Additionally, liquid chromatography–tandem mass spectrometry methods have been established to quantify SLI‐F06 concentrations in rat and pig plasma, supporting the TK studies mentioned above.

These preclinical studies collectively supported the US FDA's approval of IND application, enabling first‐in‐human studies for SLI‐F06 (ClinicalTrials.gov: NCT03880058 and NCT05501327).

## 3 Discussion

Scarring, an integral part of the body's natural healing process, occurs when the skin sustains damage from various causes such as injuries, surgery, burns, severe acne, or infections. Factors like the depth, size, location of the wound, and individual healing factors influence the scar's appearance. While scarring can impact a person's appearance and self‐esteem, posing a cosmetic issue [1, 5], it is essential to note that the implications of scarring extend far beyond aesthetics. Scars can also cause functional limitations, particularly in areas that affect mobility or other bodily functions [3, 4]. For instance, facial scars have been shown to affect psychosocial functions [32], and inelastic scar tissue following the surgical repair of a cleft lip can promote maxillary hypoplasia, necessitating complicated Le Fort I or II osteotomies to correct maxillary deficiencies [33, 34]. Currently, patients with significant scarring who seek scar revision surgery are offered two main options to minimize the high rate of excessive scar recurrence [35]: local corticosteroid injection and radiation therapy. However, these treatments often exhibit inconsistent efficacy and undesirable side effects [35]. US FDA‐approved corticosteroids, such as TAC, are used for intralesional injections into cutaneous scars, but they can lead to pigmentary changes, granulomas, skin atrophy, and reduced wound strength [36]. Meanwhile, radiation therapy is associated with growth inhibition, impaired wound strength, and an increased risk of secondary malignancies [37, 38]. Notably, decreased wound strength can lead to wound dehiscence, a common immediate postoperative complication associated with a high incidence of delayed wound healing, wound infection, and requiring costly operative repair [39]. Wound dehiscence can also increase scarring. Therefore, novel, effective wound management strategies, aimed at reducing scarring without compromising the reestablishment of wound tensile strength, are warranted.

Unlike adult skin wounds, which heal by scar formation, fetal skin wounds heal scarlessly by restoring normal skin appendages [40]. Utilizing gain‐ and loss‐of‐function models in fetal rodent wounds, we demonstrated that FMOD is required for fetal scarless skin repair [8]. Excitingly, FMOD also accelerated wound healing, reduced scarring, and increased wound tensile strength in multiple adult wounded animal models, including adult pig models that resemble human normal and HS [9, 10, 22]. Further investigation revealed a unique mechanism of action whereby a single, transient dose of FMOD finely orchestrates TGFβ1‐signaling pathways and regulates interleukin 1β trinary complex formation to elicit a more “fetal‐like” phenotype in adult dermal fibroblasts [9, 11, 16], distinct from its cell reprogramming properties requiring long‐term, continuous FMOD administration [41, 42, 43]. Overall, FMOD provides an exciting “molecular blueprint” for the new generation of antiscarring therapies.

FMODs among mammalian species share similar primary structures and 3D configurations with highly conserved encoding genes [16]. For instance, human FMOD comprises a 376 amino‐acid core protein and four *N*‐linked keratan sulfate chains. The N‐ and C‐terminal cysteine residues formed disulfide bonds to ensure the configuration of the core protein [2, 16]. Notably, FMOD directly binds to diverse molecules, such as collagen, lysyl oxidase, complement element C1q, and active TGFβs and their precursors, with some bindings mediated by multiple binding sites [12, 16, 44, 45, 46, 47, 48]. Thus, although synthetic peptide synthesis offers significant advantages over its parent protein from a cost and development standpoint, designing synthetic FMOD‐derivative active peptides presented a significant challenge given the complex interactions.

Following significant research efforts on the detailed structural analysis of the human FMOD core protein [2, 11, 16], several generations of bioactive FMOD‐derived peptides were created. This study demonstrates that the synthetic peptide SLI‐F06 functionally mimics FMOD, exhibiting comparable efficacy in stimulating dermal fibroblast migration/invasion and driving myofibroblast conversion/contraction. Similar to FMOD [9], SLI‐F06 provides a broad platform technology to significantly improve wound healing and minimize excessive scarring. Remarkedly, efficacy tests in mouse, rat, and pig wound models demonstrated that a single injection of SLI‐F06 at the time of wound closure is sufficient to improve scar outcome, including advancements in gross visual appearance, reduction of scar size, and acceleration in tensile strength reestablishment. Notably, the consensus sequence of the SLI‐F06‐responsive FMOD region is 100% conserved across human, mouse, rat, and pig, with 85% identity at aligned positions. This high degree of conservation underscores the biological relevance of these animal models to human applications. The simple single dosing with SLI‐F06 confers a significant competitive advantage over other products in development that require multiple injections [49, 50, 51] concerning patient acceptance, discomfort and compliance, physician adoption, cost of goods, and marketing strategies. Moreover, all local and systemic SLI‐F06 toxicity/safety studies required by the US FDA Division of Dermatology and Dental Products in our pre‐IND meeting showed no toxicity in rodent and pig models, as well as no genotoxicity. Collectively, these findings underscore the efficacy and safety of SLI‐F06 for human clinical trials, highlighting its potential for further development as a therapeutic to minimize excessive human scarring.

Despite the favorable efficacy and safety profile observed in preclinical models, several factors merit further consideration before advancing to human trials. First, significant development steps are anticipated to facilitate the pharmaceutical production of SLI‐F06 for human use. This particularly involves addressing the CMC regulatory component, which includes developing the drug product's sterilization, packaging, and lyophilization process. For example, additional formulation development may be necessary, such as adding mannitol as an excipient to enhance protection against moisture, improve stability, and permit crystal dispersion [52]. The effective dose and regimen for humans are also likely to be revised due to differences between animal models and human patients regarding metabolic rates, physiological differences, genetic factors, and more. However, before the path to approval and commercialization, the efficacy of SLI‐F06 requires further validation in human clinical trials involving diverse ethnic populations. Meanwhile, although not required for initial IND filing, future regulatory packages may include additional safety assessment in non‐human primates if requested by specific agencies or during later‐stage clinical development.

Meanwhile, although the current manuscript focused on developing and assessing the efficacy and safety of SLI‐F06 preclinically, important questions remain regarding the precise mechanism of action of SLI‐F06, which limits current understanding. A growing and now robust body of literature demonstrates that rapid yet transient myofibroblast activation is compatible with scarless repair in fetal wound healing [20, 53]. For instance, Lanning et al. showed that inducible myofibroblast activation drives regenerative closure without scarring in a fetal rat model [54], while Parekh and Hebda concluded in their review article that fetal fibroblasts exhibit a unique, rapid contractile phenotype with transient myofibroblast‐like activity in response to early wound cues, enabling scarless remodeling and contraction [55]. As noted earlier, our previous studies have demonstrated that FMOD alone is capable of rejuvenating adult dermal fibroblasts to adopt these fetal‐like characteristics, including rapid myofibroblast activation, function, and clearance, thereby significantly reducing scar formation [9, 11, 16]. Whether, to what extent, and precisely how SLI‐F06 recapitulates these desirable fetal traits is a critical mode‐of‐action question that we are actively pursuing. Notably, SLI‐F06 exerts its cellular effects on fibroblasts with markedly less dependence on coexisting TGFβ1 than native FMOD. Given that TGFβ1 is well known to block myofibroblast apoptosis and promote fibrosis [53, 56], the reduced reliance on TGFβ1 could also be a key contributor to SLI‐F06's superior antiscarring efficacy and warrants further investigation. Besides, we initially hypothesized that SLI‐F06's ability to operate independently of exogenous TGFβ1 might result from its capability to enhance or modulate the formation of the TGFβ receptor I (TGFβRI)/receptor II (TGFβRII) complex with endogenous TGFβ1. However, our preliminary studies using surface plasmon resonance reveal no direct binding between SLI‐F06 and these TGFβ receptors. The intriguing lack of interaction implies that SLI‐F06 may engage in alternative mechanisms yet to be discovered, opening up promising avenues for future mechanistic exploration. While the underlying mode of action demands further investigation, this innovative bioengineered distinction between SLI‐F06 and FMOD whole protein could lead to more consistently potent therapies, particularly in cases where TGFβ1 and its receptors are expressed at low levels and where exogenous TGFβ1 may not provide any benefits, such as in some cases of chronic venous ulcers and Type II diabetic wounds [57, 58, 59]. Furthermore, given that FMOD can also promote the healing of tendons and other tissues impaired by scar formation [60, 61], the benefits of SLI‐F06 may also expand to other tissues.

## 4 Conclusion

In summary, a technologically innovative and chemically synthesized FMOD peptide, namely, SLI‐F06, has been developed. This peptide retains comparable properties to the full FMOD protein, including promigration, protensile strength, and antifibrotic effects. Notably, SLI‐F06 features a simple, one‐time dosing regimen that can be administered intraoperatively, has a wide safety margin for dosing, and has shown no detectable toxicity in IND‐enabling preclinical studies. These advancements effectively tackle significant scientific, technical, manufacturing, and regulatory challenges associated with the development and commercialization of SLI‐F06.

## 5 Materials and Methods

All kits, antibodies, columns, chemicals, and reagents were purchased from Thermo Fisher Scientific (Canoga Park, CA) if not specified. FMOD production, RDF isolation and maintenance, cell proliferation, migration, invasion, contraction, gene expression, and immunocytochemical staining were conducted as described in our previous publications [9, 10, 19, 22, 62], with details provided in Supporting Information to optimize the structure and length of the main manuscript.

### 5.1 TGFβ1‐Binding FMOD Fragment Screening

From plasmid pLZZF01, multiple FMOD fragments were subcloned into the commercially available Champion pET SUMO Expression System with an N‐terminal His‐tag following the manufacturer's instructions. The sequences of these fragments were protected by the US Patent US 9,409,963 B2. Plasmid pSUMO/CAT was translated into the competent *E. coli* BL21 (DE3) as a control. Expression of the SUMO‐fused peptides was induced by 1 mM isopropyl β‐D‐1‐thiogalactopyranoside, and the yielded proteins were purified by the ProBond Purification System [19].

For TGFβ1‐binding screening, 50 µL/well 0.5 µg/mL TGFβ1 (Sigma–Aldrich, St. Louis, MO) in coating buffer was incubated in a 96‐well microtiter microplate at 4°C overnight. After blocking the nonspecific binding, 100 µL/well 1 µg/mL SUMO‐fused peptides were added and incubated for 1 h at 37°C and then overnight at 4°C. An enzyme‐linked immunosorbent assay was carried out the next day at room temperature and documented by OD450. All buffers were purchased from Bio‐Rad Laboratories (Hercules, CA).

### 5.2 TGFβ‐Binding Assay of SLI‐F06 and SLI‐F07

FMOD‐derived peptides, SLI‐F06 and SLI‐F07, were chemically synthesized by a CRO, PolyPeptide Group (Torrance, CA). Bovine serum albumin (as a negative control) and FMOD (as a positive control) were biotinylated by EZ‐Link NHS‐LC‐Biotin. 100 µL biotinylated proteins and peptides were bound to the Pierce Monomeric Avidin UltraLink Resin and packed in Pierce Spin Columns. 100 µL 100 ng/mL TGFβs (Sigma–Aldrich) was applied to the column and incubated at 4°C overnight. The unbound TGFβs were collected by centrifuge at 1000×*g* for 1 min. All procedures have followed the instructions provided by the manufacturer. After sterilization through a 0.22‐µm filter, unbound TGFβs were measured by the Mv1Lu bioassay [63].

### 5.3 Animal Models for Efficacy Assessment

Animal surgeries were performed under institutionally approved protocols (#2000‐058, #2008‐016) from the UCLA Chancellor's Animal Research Committee and in compliance with the NIH Guide for the Care and Use of Laboratory Animals. Throughout this study, no animals were sacrificed prior to the experimental endpoint, and no data points were excluded. Precise paper templates were designed to adhere to the shaved, relaxed skin to ensure accuracy and consistency in our elliptical excisions. An experienced microsurgeon carefully performed handheld scalpel excisions under an anatomical microscope, using these templates to significantly reduce experimental variability. To minimize bias, wound treatments were allocated randomly, and the investigators performing all subjective in vivo measurements were blinded to the treatment conditions. More details about the preclinical animal models and assessment methods (including scar visual appearance evaluation, tensile strength measurement, and histological staining) [9, 10, 19, 21] were also provided in Supporting Information.

### 5.4 Peptide Stability Analysis in Formulation Buffer

HPLC potency was used to assess the stability of the peptide in formulation buffer, which was developed and conducted by CRO Pyramid Laboratories Inc. (Costa Mesa, CA).

### 5.5 Preclinical Safety Assessments

All preclinical safety studies were performed by CROs in accordance with the US Department of Health and Human Services, FDA, US Code of Federal Regulations, Title 21, Part 58 and/or ISO/ICE 17025, 2005. Detailed information about these studies is available in Supporting Information, including summaries in Tables S1–S5.

### 5.6 Statistical Analysis

All statistical analyses, except the preclinical safety assessments, were conducted using Prism 10 (GraphPad, Boston, MA) in collaboration with the UCLA Statistical Biomathematical Consulting Clinic. Based on preliminary data, initial sample size estimation was performed via power analysis (*α* = 0.05, power = 0.8). Data were generally presented as mean ± standard deviation. Group comparisons for parametric data use one‐way ANOVA or a two‐sample *t*‐test, as appropriate, with repeated‐measures ANOVA for serial measurements. Nonparametric data were analyzed with the Mann–Whitney *U* and Kruskal–Wallis ANOVA tests. A *p* value of 0.05 (*) was considered a difference, while a *p* value < 0.005 (**) is defined as a statistically significant difference, following recent recommendations for stricter thresholds [64]. The CROs conducted statistical analyses of preclinical safety assessments in accordance with the relevant regulations (Supporting Information).

## Author Contributions

Zhong Zheng: conception and design of the work, data collection, data analysis and interpretation, drafting the manuscript, and critical revision of the article. Pin Ha: data collection, data analysis and interpretation, and drafting the manuscript. Chenshuang Li: data collection and critical revision of the article. Grace Xinlian Chang: data collection. Wenlu Jiang: data collection. Xiaoxiao Pang: data collection. Zhaohan Zeng: data analysis and interpretation. Elisabeth Leeflang: data analysis and interpretation. Kang Ting: conception of the work and critical revision of the article. Chia Soo: conception of the work and critical revision of the article. All authors have read and approved the final manuscript.

## Funding

This study was supported by NIH NIDCR (R44DE024692 and SB1DE026972), NIH NIAMS (R43AR063558 and R44AR064126), NIH/National Center for Advancing Translational Science (NCATS) UCLA CTSI grant (UL1TR000124), UCLA Operation Mend, UCLA Orthopaedic Hospital, UCLA Orthopaedic Hospital Research Center, and the International Orthodontics Foundation. The UCLA Center for NanoScience Institute Advanced Light Microscopy/Spectroscopy Shared Resource Facility provided confocal laser scanning microscopy services, supported by NIH Shared Instrumentation Grant S10OD025017 and NSF Major Research Instrumentation Grant CHE‐0722519.

## Ethics Statement

All animal experiments were carried out per the institutionally approved animal care protocols (2000‐058, 2008–016) from the Chancellor's Animal Research Committee at UCLA and in accordance with the NIH Guide for the Care and Use of Laboratory Animals.

## Conflicts of Interest

Drs. Zhong Zheng, Kang Ting, and Chia Soo are inventors of fibromodulin‐related patents assigned to UCLA. They are cofounders, officers, and equity holders in Scarless Laboratories Inc. and Saint Therapeutics, companies that sublicense these patents from the UC Regents. Authors Zhaohan Zeng and Elisabeth Leeflang are employees of Scarless Laboratories, Inc., but have no potential relevant financial or nonfinancial interests to disclose. The other authors declare no conflicts of interest.

## Supporting information




**Figure S1. Initial screening of putative TGFβ1‐binding regions of FMOD**. Based on the FMOD protein structural analysis, various FMOD fragments (only six are shown here) were expressed by the Champion^TM^ pET SUMO Expression System. The expression product of the control plasmid pET_SUMO/CAT was used as the negative control (N.C.), while recombinant SUMO‐fused FMOD whole protein served as the positive control. The enzyme‐linked immunosorbent assay results suggested that two FMOD regions, E and F, exhibited significant capability binding to TGFβ1. N = 3; *, *P* < 0.05; **, *P* < 0.005, respectively, compared with the negative control.
**Figure S2. TGFβ‐binding activities of synthesized FMOD‐derived peptides**. Biotinylated proteins and peptides were bound to Pierce^TM^ Monomeric Avidin UltraLink^TM^ Resin, followed by incubation with TGFβ1 (**A**), TGFβ2 (**B**), and TGFβ3 (**C**), respectively. Non‐bound TGFβs were then collected, sterilized, and quantified by relative growth inhibition of Mv1Lu cells. Data is presented as the percentage of the initially used TGFβs. N = 3; **, *P* < 0.005, compared with the negative control, biotinylated bovine serum albumin (BSA).
**Figure S3. Different effects of synthesized FMOD‐derived peptides on adult RDF proliferation**. SLI‐F06 (**A**) and SLI‐F07 (**B**) alone did not significantly affect adult RDF proliferation when applied at concentrations below 200 nM. At higher concentrations (400 nM and 800 nM), SLI‐F06 application resulted in increased RDF proliferation, while SLI‐F07 led to reduced proliferation. Although TGFβ1 alone inhibited RDF proliferation, SLI‐F06 significantly stimulated RDF proliferation in the presence of TGFβ1 (**A**). On the other hand, SLI‐F07 markedly enhanced the inhibitory effect of TGFβ1 (**B**). Data were normalized to untreated RDFs (dashed lines). N = 6; *, *P* < 0.05; **, *P* < 0.005, respectively, compared with the vehicle buffer control; ^##^, *P* < 0.005, compared with the group treated with TGFβ1 alone; and ^&&^, *P* < 0.005, comparison between the without and with TGFβ1 groups at the same SLI‐F06 (**A**) or SLI‐F07 (**B**) concentration
**Figure S4. Gene expression of adult RDFs responding to the SLI‐F06 and SLI‐F07 treatment**. Expression of *Tgfβ1* (**A**), *Ctgf* (**B**), *Acta2* (**C**), *Mmp2* (**D**), and *Fmod* (**E**) was normalized to untreated RDFs at time 0 (dashed lines). N = 3; *, *P* < 0.05; **, *P* < 0.005, respectively, compared with the vehicle buffer control; ^##^, *P* < 0.005, compared with the group treated with SLI‐F06 alone; ^&^, *P* < 0.05; ^&&^, *P* < 0.005, compared with the group treated with SLI‐F07 alone; and ^$^, *P* < 0.05; ^$$^, *P* < 0.005, compared with the group treated with TGFβ1 alone.
**Figure S5. Schematic illustration of scar index (SI) measurement**. T_D_: the distance between the epidermal‐dermal junction down to the *panniculus carnosus* (rodent models) or fascia (pig model). Adopted from our previous publication: Delayed wound closure in fibromodulin‐deficient mice is associated with increased TGF‐β3 signaling. *J Invest Dermatol* 131 (3): 769‐78. https://doi.org/10.1038/jid.2010.381.
**Figure S6. Different effects of synthesized FMOD‐derived peptides in adult mouse wound models**. Hematoxylin and eosin (H & E) staining was used to elucidate adult wildtype (WT) and *Fmod^−/−^* mouse wounds at day 14 post‐injury with the treatment of 2 mg/ml SLI‐F06 or SLI‐F07 and those who received the vehicle buffer control. Blue lines outline scars (**A**). Scar Index quantified scar size in wildtype (**B**) and *Fmod^−/−^* mice (**C**). Scale bar = 200 µm. N = 16; **, *P* < 0.005, compared with the vehicle buffer control
**Figure S7. Different effects of synthesized FMOD‐derived peptides in adult rat wound models**. H & E staining was used to elucidate adult rat wounds at day 14 post‐injury treated with vehicle buffer control, 2 mg/ml SLI‐F06 or SLI‐F07. Blue lines outline scars (**A**). Scar Index quantified scar size (**B**). Scale bar = 200 µm. N = 18; **, *P* < 0.005, compared with the vehicle buffer control.
**Figure S8. Different effects of synthesized FMOD‐derived peptides in adult Yorkshire pig wounds**. H & E staining and Masson's Trichrome staining were used to elucidate the 0.5 cm‐width x 1.5 cm‐length wounds in adult Yorkshire pigs at week 8 post‐injury with the treatment of 2 mg/ml SLI‐F06 or SLI‐F07 and those who received the vehicle buffer control. Blue (on H & E staining) and yellow (on Masson's Trichrome staining) lines outline scars (**A**). Scar Index quantified scar size (**B**). Scale bar = 500 µm. N = 6; **, *P* < 0.005, compared with the vehicle buffer control.
**Figure S9. Stability of SLI‐F06 in the formulation buffer**. HPLC was used to determine the stability of SLI‐F06 in the formulation buffer at 5°C and 25°C. 25 mg/mL SLI‐F06 in 50 mL formulation buffer was used for the testing. The testing was conducted by a CRO, pyramid Laboratories Inc.
**Table S1**: Key study design parameters of the 28‐day repeat intravenous dose toxicity study in Sprague Dawley rats with a 14‐day recovery.
**Table S2**: Key study design parameters of the 5‐day repeat subcutaneous dose toxicity study in Göttingen minipigs with a 7‐day recovery.
**Table S3**: Key study design parameters of the 3‐day repeat intradermal dose toxicity study in Göttingen minipigs with a 14‐day recovery.
**Table S4**: Animal assessment for the pulmonary safety assessment in the conscious rats.
**Table S5**: Bacterial strains and positive controls used in the Ames assay.
**Table S6**: TK parameters of 28‐day repeat intravenous dose toxicity study in Sprague Dawley rats (Table S1). *
**Table S7**: TK parameters of the 5‐Day repeat subcutaneous dose toxicity study in Göttingen minipigs (Table S2).*
**Table S8**: TK parameters of the 3‐Day repeat intradermal dose toxicity study in wounded Göttingen pigs (Table S3).*
**Table S9**: Skin irritation assay. EpiDerm reconstructed human epidermis model.
**Table S10**: Evaluation of the respiratory function of SLI‐F06 in rats (Table S4).*

## Data Availability

The data supporting the findings of this study are available within the article and its Supporting Information.

## References

[1] M. El Kinani and F. Duteille , “Scar Epidemiology and Consequences,” in Textbook on Scar Management: State of the Art Management and Emerging Technologies, ed. L. Teot , T. A. Mustoe , E. Middelkoop , and G. G. Gauglitz , (2020), 45–49.

[2] X. Pang , N. Dong , and Z. Zheng , “Small Leucine‐Rich Proteoglycans in Skin Wound Healing,” Frontiers in Pharmacology 10 (2019): 1649. 32063855 10.3389/fphar.2019.01649 PMC6997777

[3] V. L. Young and J. Hutchison , “Insights Into Patient and Clinician Concerns About Scar Appearance: Semiquantitative Structured Surveys,” Plastic and Reconstructive Surgery 124, no. 1 (2009): 256–265. 19568089 10.1097/PRS.0b013e3181a80747

[4] S. Aarabi , K. A. Bhatt , Y. Shi , et al., “Mechanical Load Initiates Hypertrophic Scar Formation Through Decreased Cellular Apoptosis,” FASEB journal: official publication of the Federation of American Societies for Experimental Biology 21, no. 12 (2007): 3250–3261. 17504973 10.1096/fj.07-8218com

[5] T. Radulesco , J. Mancini , M. Penicaud , et al., “Cross‐cultural Adaptation Into French and Validation of the SCAR‐Q Questionnaire,” Quality of Life Research 30, no. 4 (2021): 1225–1231. 33389488 10.1007/s11136-020-02719-8

[6] M. Ngaage and M. Agius , “The Psychology of Scars: A Mini‐Review,” Psychiatr Danub 30, no. Suppl 7 (2018): 633–638. 30439862

[7] U. M. Parikh , J. Mentz , I. Collier , et al., “Strategies to Minimize Surgical Scarring: Translation of Lessons Learned From Bedside to Bench and Back,” Adv Wound Care (New Rochelle) 11, no. 6 (2022): 311–329. 34416825 10.1089/wound.2021.0010

[8] Z. Zheng , X. Zhang , C. Dang , et al., “Fibromodulin Is Essential for Fetal‐type Scarless Cutaneous Wound Healing,” American Journal of Pathology 186, no. 11 (2016): 2824–2832. 27665369 10.1016/j.ajpath.2016.07.023 PMC5222972

[9] Z. Zheng , A. W. James , C. Li , et al., “Fibromodulin Reduces Scar Formation in Adult Cutaneous Wounds by Eliciting a Fetal‐Like Phenotype,” Signal Transduction And Targeted Therapy 2 (2017): 17050, Article. 29201497 10.1038/sigtrans.2017.50 PMC5661627

[10] W. Jiang , K. Ting , S. Lee , et al., “Fibromodulin Reduces Scar Size and Increases Scar Tensile Strength in Normal and Excessive‐mechanical‐loading Porcine Cutaneous Wounds,” Journal of Cellular and Molecular Medicine 22, no. 4 (2018): 2510–2513. 29392829 10.1111/jcmm.13516 PMC5867110

[11] W. Jiang , X. Pang , P. Ha , et al., “Fibromodulin Selectively Accelerates Myofibroblast Apoptosis in Cutaneous Wounds by Enhancing Interleukin 1β Signaling,” Nature Communications 16, no. 1 (2025): 3499. 10.1038/s41467-025-58906-z PMC11993684 40221432

[12] A. Hildebrand , M. Romaris , L. Rasmussen , et al., “Interaction of the Small Interstitial Proteoglycans Biglycan, Decorin and Fibromodulin With Transforming Growth Factor β,” Biochemical Journal 302, no. Pt 2 (1994): 527–534. 8093006 10.1042/bj3020527 PMC1137259

[13] S. Marqus , E. Pirogova , and T. J. Piva , “Evaluation of the Use of Therapeutic Peptides for Cancer Treatment,” Journal of Biomedical Science 24, no. 1 (2017): 21. 28320393 10.1186/s12929-017-0328-x PMC5359827

[14] M. Liu , X. Fang , Y. Yang , and C. Wang , “Peptide‐Enabled Targeted Delivery Systems for Therapeutic Applications,” Frontiers in Bioengineering and Biotechnology 9 (2021): 701504. 34277592 10.3389/fbioe.2021.701504 PMC8281044

[15] S. Beheshtirouy , F. Mirzaei , S. Eyvazi , and V. Tarhriz , “Recent Advances in Therapeutic Peptides for Breast Cancer Treatment,” Current Protein & Peptide Science 22, no. 1 (2021): 74–88. 33208071 10.2174/1389203721999201117123616

[16] Z. Zheng , H. S. Granado , and C. Li , “Fibromodulin, a Multifunctional Matricellular Modulator,” Journal of Dental Research 102, no. 2 (2023): 125–134. 36515321 10.1177/00220345221138525 PMC9986681

[17] J. G. Marblestone , S. C. Edavettal , Y. Lim , P. Lim , X. Zuo , and T. R. Butt , “Comparison of SUMO Fusion Technology With Traditional Gene Fusion Systems: Enhanced Expression and Solubility With SUMO,” Protein Science 15, no. 1 (2006): 182–189. 16322573 10.1110/ps.051812706 PMC2242369

[18] Solability Guidlines for Peptides , https://www.sigmaaldrich.com/US/en/technical‐documents/technical‐article/research‐and‐disease‐areas/cell‐and‐developmental‐biology‐research/solubility‐guidelines.

[19] Z. Zheng , C. Nguyen , X. Zhang , et al., “Delayed Wound Closure in Fibromodulin‐deficient Mice Is Associated With Increased TGF‐beta3 Signaling,” The Journal of investigative dermatology 131, no. 3 (2011): 769–778. 21191417 10.1038/jid.2010.381 PMC4073663

[20] M. L. Bochaton‐Piallat , G. Gabbiani , and B. Hinz , “The Myofibroblast in Wound Healing and Fibrosis: Answered and Unanswered Questions,” F1000Research 5 (2016): 752. 10.12688/f1000research.8190.1 PMC4847562 27158462

[21] H. Khorasani , Z. Zheng , C. Nguyen , et al., “A Quantitative Approach to Scar Analysis,” American Journal of Pathology 178 (2011): 621–628. 21281794 10.1016/j.ajpath.2010.10.019 PMC3070584

[22] Z. Zheng , K. S. Lee , X. Zhang , et al., “Fibromodulin‐deficiency Alters Temporospatial Expression Patterns of Transforming Growth Factor‐beta Ligands and Receptors During Adult Mouse Skin Wound Healing,” PLoS ONE 9, no. 6 (2014): e90817. 24603701 10.1371/journal.pone.0090817 PMC3948369

[23] Z. Zheng , J. Jian , O. Velasco , et al., “Fibromodulin Enhances Angiogenesis During Cutaneous Wound Healing,” Plastic and reconstructive surgery Global open 2, no. 12 (2015): e275. 25587509 10.1097/GOX.0000000000000243 PMC4292257

[24] B. Kollerup Madsen , M. Hilscher , D. Zetner , and J. Rosenberg , “Adverse Reactions of Dimethyl Sulfoxide in Humans: A Systematic Review,” F1000Research 7 (2018): 1746. 31489176 10.12688/f1000research.16642.1 PMC6707402

[25] M. Lambros , T. H. Tran , Q. Fei , and M. Nicolaou , “Citric Acid: A Multifunctional Pharmaceutical Excipient,” Pharmaceutics 14, no. 5 (2022): 972. 35631557 10.3390/pharmaceutics14050972 PMC9148065

[26] M. Sheng , Y. Chen , H. Li , Y. Zhang , and Z. Zhang , “The Application of Corticosteroids for Pathological Scar Prevention and Treatment: Current Review and Update,” Burns Trauma 11 (2023): tkad009. 36950503 10.1093/burnst/tkad009 PMC10025010

[27] I. A. Darby , B. Laverdet , F. Bonte , and A. Desmouliere , “Fibroblasts and Myofibroblasts in Wound Healing,” Clinical, cosmetic and investigational dermatology 7 (2014): 301–311. 25395868 10.2147/CCID.S50046 PMC4226391

[28] G. C. Gurtner , R. H. Dauskardt , V. W. Wong , et al., “Improving Cutaneous Scar Formation by Controlling the Mechanical Environment: Large Animal and Phase I Studies,” Annals of Surgery 254, no. 2 (2011): 217–225. 21606834 10.1097/SLA.0b013e318220b159

[29] Y. Y. Elsayed , T. Kuhl , and D. Imhof , “Regulatory Guidelines for the Analysis of Therapeutic Peptides and Proteins,” Journal of Peptide Science 31, no. 3 (2025): e70001. 39921384 10.1002/psc.70001 PMC11806371

[30] How to Evaluate the Quality of Custom Synthetic Peptides? Mtoz Biolabs, https://www.mtoz‐biolabs.com/how‐to‐evaluate‐the‐quality‐of‐custom‐synthetic‐peptides.html.

[31] Q5C Quality of Biotechnological Products: Stability Testing of Biotechnological/Biological Products (International Conference on Harmonisation) (1996).

[32] N. Ziolkowski , S. C. Kitto , D. Jeong , et al., “Psychosocial and Quality of Life Impact of Scars in the Surgical, Traumatic and Burn Populations: A Scoping Review Protocol,” BMJ Open 9, no. 6 (2019): e021289. 10.1136/bmjopen-2017-021289 PMC6561410 31164358

[33] A. Rachmiel , M. Even‐Almos , and D. Aizenbud , “Treatment of Maxillary Cleft Palate: Distraction Osteogenesis vs. orthognathic Surgery,” Annals of Maxillofacial Surgery 2, no. 2 (2012): 127–130. 23483803 10.4103/2231-0746.101336 PMC3591052

[34] S. Abuzinada and A. Alyamani , “Stability of Maxillary Advancement Using External Rigid Distractors in Cleft Lip and Palate Patients,” Open Journal of Stomatology 2 (2012): 383–387.

[35] G. G. Gauglitz , H. C. Korting , T. Pavicic , T. Ruzicka , and M. G. Jeschke , “Hypertrophic Scarring and Keloids: Pathomechanisms and Current and Emerging Treatment Strategies,” Molecular Medicine 17, no. 1‐2 (2011): 113–125. 20927486 10.2119/molmed.2009.00153 PMC3022978

[36] E. J. Feldman , “Cutaneous Reactions to Corticosteroids,” in Cutaneous Drug Eruptions: Diagnosis, Histopathology and Therapy, ed. JC Hall and BJ Hall (Springer London, 2015), 353–359.

[37] F. Haubner , E. Ohmann , F. Pohl , J. Strutz , and H. G. Gassner , “Wound Healing After Radiation Therapy: Review of the Literature,” Radiation oncology 7 (2012): 162. 23006548 10.1186/1748-717X-7-162 PMC3504517

[38] G. Juckett and H. Hartman‐Adams , “Management of Keloids and Hypertrophic Scars,” American Family Physician 80 (2009): 253–260. 19621835

[39] R. D. Rosen and B. Manna , “Wound Dehiscence,” StatPearls (2023). 31869176

[40] B. J. Larson , M. T. Longaker , and H. P. Lorenz , “Scarless Fetal Wound Healing: A Basic Science Review,” Plastic and Reconstructive Surgery 126, no. 4 (2010): 1172–1180. 20885241 10.1097/PRS.0b013e3181eae781 PMC4229131

[41] Z. Zheng , J. Jian , X. Zhang , et al., “Reprogramming of human Fibroblasts Into Multipotent Cells With a Single ECM Proteoglycan, Fibromodulin,” Biomaterials 33, no. 24 (2012): 5821–5831. 22622142 10.1016/j.biomaterials.2012.04.049

[42] Z. Zheng , C. Li , P. Ha , et al., “ *CDKN2B* upregulation Prevents Teratoma Formation in Multipotent Fibromodulin Reprogrammed Cells,” Journal of Clinical Investigation 129, no. 8 (2019): 3236–3251. 31305260 10.1172/JCI125015 PMC6668700

[43] P. Yang , C. Li , M. Lee , et al., “Photopolymerizable Hydrogel‐Encapsulated Fibromodulin‐Reprogrammed Cells for Muscle Regeneration,” Tissue Engineering Part A 26, no. 19‐20 (2020): 1112–1122. 32323608 10.1089/ten.tea.2020.0026 PMC7580647

[44] S. Kalamajski and A. Oldberg , “The Role of Small Leucine‐rich Proteoglycans in Collagen Fibrillogenesis,” Matrix Biology 29, no. 4 (2010): 248–253. 20080181 10.1016/j.matbio.2010.01.001

[45] S. Kalamajski , D. Bihan , A. Bonna , K. Rubin , and R. W. Farndale , “Fibromodulin Interacts With Collagen Cross‐linking Sites and Activates Lysyl Oxidase,” The Journal of Biological Chemistry 291, no. 15 (2016): 7951–7960. 26893379 10.1074/jbc.M115.693408 PMC4825002

[46] J. Zeng‐Brouwers , S. Pandey , J. Trebicka , M. Wygrecka , and L. Schaefer , “Communications via the Small Leucine‐rich Proteoglycans: Molecular Specificity in Inflammation and Autoimmune Diseases,” Journal of Histochemistry and Cytochemistry 68, no. 12 (2020): 887–906. 32623933 10.1369/0022155420930303 PMC7708667

[47] N. Burton‐Wurster , W. Liu , G. L. Matthews , et al., “TGF Beta 1 and Biglycan, Decorin, and Fibromodulin Metabolism in Canine Cartilage,” Osteoarthritis and Cartilage 11, no. 3 (2003): 167–176. 12623288 10.1053/s1063-4584(02)00349-7

[48] V. Tillgren , M. Morgelin , P. Onnerfjord , S. Kalamajski , and A. Aspberg , “The Tyrosine Sulfate Domain of Fibromodulin Binds Collagen and Enhances Fibril Formation,” The Journal of biological chemistry 291, no. 45 (2016): 23744–23755. 27634037 10.1074/jbc.M116.730325 PMC5095427

[49] P. Pavco , L. Libertine , V. L. Young , N. G. Paz , J. Hunstad , and G. Cauwenbergh , “Update on Phase 2 Clinical Trial Results of RXI‐109 Treatment to Reduce the Formation of Hypertrophic Dermal Scars,” Journal of the American Academy of Dermatology 72, no. 5 (2015): Ab273–Ab273.

[50] J. Jensen , G. Gentzkow , G. Berman , et al., “Anti‐CTGF Oligonucleotide Reduces Severity of Postsurgical Hypertrophic Scars in a Randomized, Double‐Blind, Within‐Subject, Placebo‐Controlled Study,” Plastic and Reconstructive Surgery 142, no. 2 (2018): 192e–201e. 10.1097/PRS.0000000000004590 30045185

[51] S. S. Qiu , J. Dotor , and B. Hontanilla , “Effect of P144(R) (Anti‐TGF‐beta) in an “in Vivo” Human Hypertrophic Scar Model in Nude Mice,” PLoS ONE 10, no. 12 (2015): e0144489. 26720517 10.1371/journal.pone.0144489 PMC4697841

[52] H. L. Ohrem , E. Schornick , A. Kalivoda , and R. Ognibene , “Why Is Mannitol Becoming More and More Popular as a Pharmaceutical Excipient in Solid Dosage Forms?,” Pharmaceutical Development and Technology 19, no. 3 (2014): 257–262. 23528124 10.3109/10837450.2013.775154

[53] Y. Tai , E. L. Woods , J. Dally , et al., “Myofibroblasts: Function, Formation, and Scope of Molecular Therapies for Skin Fibrosis,” Biomolecules 11, no. 8 (2021): 1095. 34439762 10.3390/biom11081095 PMC8391320

[54] D. A. Lanning , R. F. Diegelmann , D. R. Yager , M. L. Wallace , C. E. Bagwell , and J. H. Haynes , “Myofibroblast Induction With Transforming Growth Factor‐beta1 and ‐beta3 in Cutaneous Fetal Excisional Wounds,” Journal of Pediatric Surgery 35, no. 2 (2000): 183–187, discussion 187–8. 10693663 10.1016/s0022-3468(00)90007-1

[55] A. Parekh and P. A. Hebda , “The Contractile Phenotype of Dermal Fetal Fibroblasts in Scarless Wound Healing,” Curr Pathobiol Rep 5, no. 3 (2017): 271–277. 29038745 10.1007/s40139-017-0149-3 PMC5640269

[56] B. Hinz and D. Lagares , “Evasion of Apoptosis by Myofibroblasts: A Hallmark of Fibrotic Diseases,” Nat Rev Rheumatol 16, no. 1 (2020): 11–31. 31792399 10.1038/s41584-019-0324-5 PMC7913072

[57] I. Pastar , O. Stojadinovic , A. Krzyzanowska , et al., “Attenuation of the Transforming Growth Factor Beta‐signaling Pathway in Chronic Venous Ulcers,” Molecular Medicine 16, no. 3‐4 (2010): 92–101. 20069132 10.2119/molmed.2009.00149 PMC2804290

[58] A. J. Cowin , N. Hatzirodos , C. A. Holding , et al., “Effect of Healing on the Expression of Transforming Growth Factor Beta(s) and Their Receptors in Chronic Venous Leg Ulcers,” The Journal of investigative dermatology 117, no. 5 (2001): 1282–1289. 11710945 10.1046/j.0022-202x.2001.01501.x

[59] F. Al‐Mulla , S. J. Leibovich , I. M. Francis , and M. S. Bitar , “Impaired TGF‐beta Signaling and a Defect in Resolution of Inflammation Contribute to Delayed Wound Healing in a Female Rat Model of Type 2 Diabetes,” Molecular Biosystems 7, no. 11 (2011): 3006–3020. 21850315 10.1039/c0mb00317d

[60] X. Xu , P. Ha , E. Yen , C. Li , and Z. Zheng , “Small Leucine‐Rich Proteoglycans in Tendon Wound Healing,” Adv Wound Care (New Rochelle) 11, no. 4 (2022): 202–214. 34978952 10.1089/wound.2021.0069

[61] X. Xu , Y. Zhang , P. Ha , et al., “A Novel Injectable Fibromodulin‐releasing Granular Hydrogel for Tendon Healing and Functional Recovery,” Bioeng Transl Med 8, no. 1 (2023): e10355. 36684085 10.1002/btm2.10355 PMC9842059

[62] C. S. Li , P. Yang , K. Ting , et al., “Fibromodulin Reprogrammed Cells: A Novel Cell Source for Bone Regeneration,” Biomaterials 83 (2016): 194–206. 26774565 10.1016/j.biomaterials.2016.01.013 PMC4754141

[63] A. Meager , “Assays for Transforming Growth Factor Beta,” Journal of Immunological Methods 141, no. 1 (1991): 1–14. 1865116 10.1016/0022-1759(91)90204-s

[64] D. J. Benjamin , J. O. Berger , M. Johannesson , et al., “Redefine Statistical Significance. We Propose to Change the Default *p‐*value Threshold for Statistical Significance From 0.05 to 0.005 for Claims of New Discoveries,” Nature Human Behaviour 2 (2018): 6–10. 10.1038/s41562-017-0189-z 30980045

