# Supplementary material for: Preclinical Efficacy and Safety Study of a Novel Dermal Fibroblast Modulating Drug, SLI‐F06, in Cutaneous Wound Healing
Source: MedComm (2020). 2026 May 24;7(6):e70761. doi: 10.1002/mco2.70761 (PMC13239954; doi:10.1002/mco2.70761)
Supplement: Supplementary file 1 — Figure S1. Initial screening of putative TGFβ1‐binding regions of FMOD. Based on the FMOD protein structural analysis, various FMOD fragments (only six are shown here) were expressed by the ChampionTM pET SUMO Expression System. The expression product of the control plasmid pET_SUMO/CAT was used as the negative control (N.C.), while recombinant SUMO‐fused FMOD whole protein served as the positive control. The enzyme‐linked immunosorbent assay results suggested that two FMOD regions, E and F, exhibited significant capability binding to TGFβ1. N = 3; *, P < 0.05; **, P < 0.005, respectively, compared with the negative control. Figure S2. TGFβ‐binding activities of synthesized FMOD‐derived peptides. Biotinylated proteins and peptides were bound to PierceTM Monomeric Avidin UltraLinkTM Resin, followed by incubation with TGFβ1 (A), TGFβ2 (B), and TGFβ3 (C), respectively. Non‐bound TGFβs were then collected, sterilized, and quantified by relative growth inhibition of Mv1Lu cells. Data is presented as the percentage of the initially used TGFβs. N = 3; **, P < 0.005, compared with the negative control, biotinylated bovine serum albumin (BSA). Figure S3. Different effects of synthesized FMOD‐derived peptides on adult RDF proliferation. SLI‐F06 (A) and SLI‐F07 (B) alone did not significantly affect adult RDF proliferation when applied at concentrations below 200 nM. At higher concentrations (400 nM and 800 nM), SLI‐F06 application resulted in increased RDF proliferation, while SLI‐F07 led to reduced proliferation. Although TGFβ1 alone inhibited RDF proliferation, SLI‐F06 significantly stimulated RDF proliferation in the presence of TGFβ1 (A). On the other hand, SLI‐F07 markedly enhanced the inhibitory effect of TGFβ1 (B). Data were normalized to untreated RDFs (dashed lines). N = 6; *, P < 0.05; **, P < 0.005, respectively, compared with the vehicle buffer control; ##, P < 0.005, compared with the group treated with TGFβ1 alone; and &&, P < 0.005, comparison between the witho [file MCO2-7-e70761-s001.docx]

**Supplementary Materials**

**Title:**

Preclinical efficacy and safety study of a novel dermal fibroblast modulating drug, SLI-F06, in cutaneous wound healing

**Running Title:**

Preclinical efficacy and safety study of SLI-F06

**Authors:**

Zhong Zheng,^1,2,#,*^ Pin Ha,^3,4,#^ Chenshuang Li,^5^ Grace Xinlian Chang,^6^ Wenlu Jiang,^3^ Xiaoxiao Pang,^7^ Zhaohan Zeng,^1^ Elisabeth Leeflang,^1^ Kang Ting,^1,8,*^ Chia Soo^1,3,4,9,*^

1. Scarless Laboratories, Inc., Torrance, CA 90502, USA
2. Department of Periodontics, School of Dental Medicine, University of Pennsylvania, Philadelphia, PA 19104, USA
3. Division of Plastic and Reconstructive Surgery, Department of Surgery, David Geffen School of Medicine, University of California, Los Angeles, Los Angeles, CA 90095, USA
4. Department of Orthopaedic Surgery and the Orthopaedic Hospital Research Center, David Geffen School of Medicine, University of California, Los Angeles, Los Angeles, CA 90095, USA
5. Department of Orthodontics, School of Dental Medicine, University of Pennsylvania, Philadelphia, PA 19104, USA
6. Department of Surgery, David Geffen School of Medicine, University of California, Los Angeles, Los Angeles, CA 90095, USA
7. Chongqing Key Laboratory of Oral Diseases and Biomedical Sciences, Chongqing Municipal Key Laboratory of Oral, Biomedical Engineering of Higher Education, Stomatological Hospital of Chongqing Medical University, Chongqing 401147, P. R. China,
8. American Dental Association Forsyth Institute, Cambridge, MA 02142, USA
9. Department of Bioengineering, Henry Samueli School of Engineering and Applied Science, University of California, Los Angeles, Los Angeles, CA 90095, USA

^#^ Co-first authors

*** Senior and Corresponding authors:**

Zhong Zheng

1124 W Carson St. – Biolabs at Lundquist

MRL Building, 3^rd^ Floor

Torrance, CA 90502, USA

Phone: +1-(323)-496-6649

Email: [leozz95@scarlesslabs.com](mailto:leozz95@scarlesslabs.com)

Kang Ting

American Dental Association Forsyth Institute

Cambridge, MA 02142, USA

Phone: +1-(310)-713-9979

Email: [erickangting@gmail.com](mailto:erickangting@gmail.com)

Chia Soo

MRL 2641a

675 Charles E Young Drive South,

Los Angeles, CA 90095, USA

Phone: +1-(310)-713-9978

Email: [bsoo@g.ucla.edu](mailto:bsoo@g.ucla.edu)

**Supplementary Materials and Methods**

**Fibromodulin (FMOD) production**

A cDNA encoding human FMOD (GenBank accession number: NM_002023) was subcloned into the pSecTag2A vector to generate a C-terminal His-tagged construct. This plasmid, designated pLZZF01, was then transfected into CHO-K1 cells (ATCC, Manassas, VA)^1^. The production and purification of FMOD were conducted by a contract research organization (CRO), GenScript (Piscataway, NJ). Briefly, a stable CHO-K1 cell line engineered to express FMOD was cultured in a 1L bioreactor under standard conditions (37°C, 5% CO₂), using serum-free Freestyle CHO Expression Medium. Following the culture period, cell culture supernatant was harvested on day 10 for purification using a HiTrap^TM^ IMAC HP 1-mL column (GE Healthcare, Uppsala, Sweden). Following elution with 100 mM imidazole, the fractions were dialyzed against 20 mM phosphate-buffered saline (PBS), pH 7.4. The resulting low-conductivity sample was subsequently applied to a HiTrap^TM^Q HP 1-mL column (GE Healthcare) for further purification. Finally, FMOD was purified under non-reducing conditions, followed by dialysis as previously described^2^ and lyophilization. Prior to use, FMOD was reconstituted in PBS and sterilized by filtration through a 0.22-μm filter.

**Rat dermal fibroblast (RDF) isolation and maintenance**

Adult RDFs were isolated from the dorsal skin of adult male Sprague-Dawley rats and cultured in Dulbecco's Modified Eagle's Medium (DMEM) with 1% (v/v) penicillin/streptomycin and 10% (v/v) fetal bovine serum (FBS)^3,4^. The cells tested negative for mycoplasma (Universal Mycoplasma Detection Kit; ATCC) and were used at passage 3 for all *in vitro* tests^4^.

**Cell proliferation assay**

RDFs were seeded in 96-well plates at a density of 2 x 10^3^ cells/well. To synchronize the cell cycle and eliminate the effects of residual growth factors, cells were serum-starved for 16 hours prior to treatment. Subsequently, cells were treated with 100 μl fresh medium containing tested peptides and/or 100 pM transforming growth factor (TGF)β1. Following a 48-h incubation, cell proliferation was measured by the Click-iT EdU Proliferation Assay for Microplates^4^.

**Cell migration assay**

Following 16 h of serum starvation, scratches approximately 1 mm wide were created in the RDF monolayers in 6-well plates using a sterile pipette tip. To ensure consistency, scratch widths were verified microscopically immediately after wounding, and only those measuring 1.0 ± 0.1 mm were selected for the study. After washing three times with PBS to remove detached cells, the wounded monolayers were incubated in the treatment medium: DMEM ± 200 nM of the tested peptides ± 100 pM of TGFβ1 or TGFβ3^1^. Following a 24-h incubation, cells were fixed in cold methanol for 5 min and photographed. Cell migration was quantified as the average wound gap between the wound edges with Image-Pro^®^ Plus 6.0 software (Media Cybernetics, Rockville, MD).

**Cell invasion assay**

Cell invasion was assessed using FluoroBlok^TM^ HTS inserts (8-μm pore size, Fluorescence Blocking PET track-etched membranes; BD Biosciences, Franklin Lakes, NJ) in 24-well tissue culture plates. The inserts were coated with 200 μl of collagen, rinsed with DMEM, and assembled into wells containing 500 μl treatment medium (described above). Serum-starved RDFs (2 x 10^4^ cells in 100 μl treatment medium) were then added to the inserts. Following a 24-h incubation, the upper surfaces of the insert membranes were swabbed to remove non-invaded cells. Cells that had moved to the lower surface were fixed in cold methanol, stained with 4',6-diamidino-2-phenylindole (DAPI; 3 μM), and quantified^1^.

**Immunocytochemical staining**

After seeding at 1 x 10^4^ cells/well in 4-well Lab-TEK^®^ II chamber slides, RDFs were serum-starved for 16 h and then treated for 48 h before processing for immunostaining^4^. Anti-α-smooth muscle actin (α-SMA) antibody and Phalloidin-iFluor 488 reagent (for F-actin staining) were purchased from Abcam (Boston, MA), while DAPI was used for counterstaining. Confocal laser scanning microscopy images were obtained by a Leica TCS-SP2-AOBS confocal microscope (Leica Microsystems, Buffalo Grove, IL).

**Collagen-based cell contraction assay**

A collagen-based contraction assay was performed using a commercial kit (Cell Contraction Assay, Cell Biolabs). Following a 16-hour serum starvation, 2 x 10^6^ RDFs/well were embedded in a 3D collagen lattice in a 24-well plate according to the manufacturer’s instructions. After collagen polymerization, 1.0 ml of treatment medium was added, and the cultures were incubated for 48 h to allow stress development. The gels were then gently released from the well walls with a sterile spatula, and the change in gel size was measured at 0, 20, 40, and 60 min post-release^4^.

**Cellular gene expression assay**

For qRT-PCR analysis, 1 x 10^5^ RDFs were placed in 10-cm tissue culture dishes, synchronized by 16-h serum starvation, and incubated in treatment medium. RNA was isolated with the RNeasy^®^ Mini Kit (Qiagen; Beverly, MA), including a DNase treatment step, and reverse-transcribed into cDNA using SuperScript^TM^ III First-Strand Synthesis System. Gene expression was quantified by qRT-PCR on a QuantStudio^TM^ 3 Real-time PCR System using pre-designed Taqman^TM^ Real-Time PCR Assays, with *glyceraldehyde-3-phosphate dehydrogenase* *(Gapdh)* as an endogenous control^4,5^. Reactions were run with three independent sets of cDNA template, and relative expression was calculated using the 2^ΔΔCT^ method^1,4,6^.

**Adult mouse skin wound model**

A full-thickness excisional wound model was established in three-month-old male 129/sv wildtype and *Fmod-null* (*Fmod^-/-^*; B6;129-FMOD<tm1Aol>/SooJ; Jackson Laboratory, Bar Harbor, ME). Following anesthesia and dorsal skin preparation, four 10 mm x 3 mm skin ellipses, including the *panniculus carnosus*, were excised per mouse. Wounds were separated by at least 2 cm to prevent cross-effects. Each open wound edge was injected with 25 μl of the tested peptides at 2 mg/ml in PBS with 5% dimethyl sulfoxide (DMSO) as solubilizer (25 μl x 2 edges = 50 μl total/wound). The vehicle solution served as the control. 5-0 nylon sutures placed at one-third intervals were used to primarily close the wounds and removed on day 7 post-operatively. Wound samples were collected on day 14^1,4,7^.

**Adult rat skin wound model**

Skin wounds were generated on adult male Sprague-Dawley rats (~300 g; Charles River Laboratories, Ashland, OH). Under anesthesia, six full-thickness 10 x 3 mm skin ellipses (including the *panniculus carnosus*) were excised from the sterilely prepared dorsum. All wounds were spaced at least 2 cm apart. Test peptides (2 mg/mL) or vehicle control were administered *via* intradermal injection (25 µL per wound edge; 50 μl total/wound). Wounds were demarcated with permanent dye and closed primarily with 4-0 Nylon suture with one-third intervals. The sutures were removed after 7 days, and wound samples were collected 14 days post-injury^4^.

**Porcine wound healing model**

Full-thickness excisional wounds were generated on 20-kg female Yorkshire pigs (Premier BioSource, formerly S&S Farms, Ramona, CA) or red Duroc pigs (Pork Power Farms, Turlock, CA) under general anesthesia [induced by Telazol^®^ (5 mg/kg; Zoetis, Parsippany, NJ), maintained with isoflurane (at 0.5-2.5% in room air). The skin was prepared sterilely, and elliptical wounds (1.5 cm length, with 0.5 cm or 2.0 cm widths) were created with a #15 surgical blade down to the fascia, oriented along the anterior-posterior axis (parallel to the head-to-tail direction of the animal), and spaced at least 2 cm apart. A total of 24 wounds were created per animal. At surgery, each wound edge was injected with 100 μl treatment (100 μl x 2 edges = 200 μl total/wound). Marked with permanent dye, wounds were primarily closed with 3-0 Nylon mattress sutures. Twenty-four hours later, an additional 50 μl of treatment was injected at each edge for the two-dose injection assessment. Sutures were removed 14 days post-injury, and wounds were collected at 8 weeks post-injury^4,8^.

**Scar visual appearance evaluation**

Scar quality was evaluated using an adapted Visual Analogue Score (VAS)^4,8,9^ in a randomized, blinded manner. Standardized digital photographs were taken of all scars under consistent lighting and camera settings by a highly sensitive digital camera (DSLR DS126181; Canon, Tokyo, Japan). Three experienced medical doctor assessors rated each scar for a maximum of 20 s by marking a 100-mm horizontal line, with 0 representing unwounded skin and 100 representing the poorest-quality scar (raised, hyperpigmented, or red with low acceptability and observer comfort). To ensure independent assessment, the evaluators reviewed the images simultaneously in separate rooms without communication, and another researcher collected the evaluation sheets, measured the VAS scores, and documented the average score of three assessors independently to produce the final VAS score for further statistical analysis^4,8^.

.

**Tensile strength measurement**

For tensile strength measurement, a full-thickness skin strip (4 cm x 1 cm) was dissected and mounted on an Instron 5565 Universal Testing Machine (Instron, High Wycombe, UK). Samples were clamped using pneumatic grips on a 1 cm^2^ skin area of either side of the wound to avoid specimen slippage. The force failure (breaking strength) was measured in Newtons (N)^4,8^.

**Histological staining**

Tissue processing and analysis were performed using a standardized sampling protocol to ensure reproducibility. After 24-hour fixation in 10% neutral-buffered formalin, samples were dehydrated and bisected centrally, perpendicular to the wound's long axis. The central region of each half was embedded for sectioning. Serial sections were cut at 5 μm for hematoxylin and eosin (H&E) and Masson's trichrome staining, and at 10μm for Picrosirius red (PSR) staining-coupled polarized light microscopy (PLM). To obtain representative data, sectioning began from the bisected face, and every 10^th^ section was sampled for analysis. Scar area was quantified and normalized to dermal thickness using the Scar Index (**Figure S5**)^1,4,7,8^.

**Twenty-eight-day repeat intravenous dose toxicity study of SLI-F06 in Sprague-Dawley rats with a 14-day recovery period**

The CRO, Toxikon Corporation, conducted Good Laboratory Practice (GLP)-compliant systemic toxicity and toxicokinetic (TK) studies of SLI-F06 following daily intravenous administration for 28 days, with a 14-day recovery phase. Adult Sprague-Dawley rats were randomized into four groups (**Table S1**). Main necropsy occurred on Day 28, and recovery animals were sacrificed on Day 43, with remaining animals allocated to TK analysis. Endpoint evaluation included clinical observations, body weight and food intake, ophthalmology, functional observational battery, clinical pathology, organ weight, gross observations, and histopathology.

**Five-day study of SLI-F06 by subcutaneous injection in minipigs with a 7-day recovery period**

The CRO, Charles River Laboratories Inc., conducted the GLP-compliant study to assess toxicity, reversibility, and TK of SLI-F06 following once-daily subcutaneous administration for 5 days with a 7-day recovery period. Four-month-old Göttingen minipigs were randomized by body weight using a stratified randomization schedule (**Table S2**). Animals were manually restrained without sedation, and doses were administered at a consistent clipped dorsal site. Assessments included clinical signs, body weights, dermal scoring, electrocardiology, clinical pathology, TK, necropsy, organ weights, and histopathology.

**Three-day study of SLI-F06 by intradermal injection in wounded pigs with a 14-day recovery period**

The GLP-compliant study, conducted by the CRO, Charles River Laboratories Inc., evaluated intradermal toxicity and TK of SLI-F06 in wounded Göttingen minipigs. Animals were randomized by body weight (**Table S3**). With anesthesia, six 3 cm-long full-thickness dorsal incisions were created and sutured, while the doses were administered intradermally once daily for 3 days. The same endpoints as the 5-day subcutaneous study were assessed.

**Pulmonary safety assessment in the conscious rats**

Respiratory effects of intravenously administered SLI-F06 were evaluated using head-out plethysmography by Calvert Labs (Scott Township, PA) with GLP compliance. Dosing schedules are summarized in **Table S4**. Respiratory parameters, including respiratory rate, tidal volume, and minute volume, were measured up to 4 h post-dose and analyzed versus vehicle and baseline controls using a two-way repeated measures ANOVA followed by a Bonferroni Multiple Comparison Test (SigmaStat, v. 2.03; Systat, Palo Alto, CA).

***Bacterial reverse mutation (Ames) test***

Toxikon Corporation assessed the potential mutagenicity of SLI-F06 in *Salmonella typhimurium* (TA98, TA100, TA1535, and TA1537) and *Escherichia coli* (WP2 *uvrA*) strains with and without metabolic activation. The formulation buffer was used as a negative control, and positive controls were listed in **Table S5** based on the appropriate solvent. All controls and test groups were plated in triplicate, and revertant colonies were counted after incubation at 37°C and compared to the number of spontaneous revertants in a negative control culture.

***In vitro* skin irritation: reconstructed human Epidermis test**

The GLP-compliant study, conducted by Toxikon Corporation, determined the irritation potential of SLI-F06 using human epidermal model EpiDerm^TM^ (EPl-200; surface area 0.63 cm^2^; MatTek, Ashland, MA). After overnight pre-incubation, tissues were topically exposed to 25 mg SLI-F06, 30 μl 5% sodium dodecyl sulfate (positive control), or Dulbecco's PBS (negative control) for 1 h at 37^o^C, and rinsed thoroughly. MTT assays were performed after the tissues were transferred into a fresh medium. Relative cell viability was calculated for each tissue as % of the mean of the negative control tissues. The skin irritation potential of the test material was predicted if the remaining relative cell viability was below 50%.

**Supplementary Tables**

**Table S1. Key study design parameters of the 28-day repeat intravenous dose toxicity study in Sprague Dawley rats with a 14-day recovery.**

| **Group** | **Dose (mg/kg/day)** | **Main Sacrifice (Day 28)** | **Recovery Sacrifice (Day 43)** | **TK** |
| --- | --- | --- | --- | --- |
| Formulation buffer control | 0 | 10F/10M* | 5F/5M | 3F/3M |
| Low-Dose | 50 | 10F/10M | 0 | 6F/6M |
| Mid-Dose | 90 | 10F/10M | 5F/5M | 6F/6M |
| High-Dose (MFD**) | 125 | 10F/10M | 5F/5M | 6F/6M |

* F, female; M, male.

** MFD, maximum feasible dose.

**Table S2. Key study design parameters of the 5-day repeat subcutaneous dose toxicity study in Göttingen minipigs with a 7-day recovery.**

| **Group** | **Dose (mg/kg/day)** | **Main Sacrifice (Day 5)** | **Recovery Sacrifice (Day 12)** |
| --- | --- | --- | --- |
| Formulation buffer control | 0 | 3F/3M* | 2F/2M |
| Low-Dose | 10 | 3F/3M | 0 |
| Mid-Dose | 78 | 3F/3M | 0 |
| High-Dose (MFD**) | 125 | 3F/3M | 2F/2M |

* F, female; M, male.

** MFD, maximum feasible dose.

**Table S3. Key study design parameters of the 3-day repeat intradermal dose toxicity study in Göttingen minipigs with a 14-day recovery.**

| **Group** | **Dose (mg/day)** | **Main Sacrifice (Day 3)** | **Recovery Sacrifice (Day 17)** |
| --- | --- | --- | --- |
| Untreated | 0 | 3F/3M* | 2F/2M |
| Formulation buffer control | 0 | 3F/3M | 2F/2M |
| Low-Dose | 24 | 3F/3M | 2F/2M |
| High-Dose (MFD)** | 60 | 3F/3M | 2F/2M |

* F, female; M, male.

** MFD, maximum feasible dose.

**Table S4. Animal assessment for the pulmonary safety assessment in the conscious rats.**

| **Group** | **Number of Animals/Sex** | **Dose (mg/kg/day)** |
| --- | --- | --- |
| Formulation buffer control | 6M* | 0 |
| Low-Dose | 6M | 50 |
| Mid-Dose | 6M | 90 |
| High-Dose (MFD)** | 6M | 125 |

* M, male.

** MFD, maximum feasible dose.

**Table S5. Bacterial strains and positive controls used in the Ames assay.**

| **Strain and Designation** | | **Strain-specific Positive Control per metabolic activation** | | | |
| --- | --- | --- | --- | --- | --- |
|  |  | **Without metabolic activation** | | **With metabolic activation** | |
|  |  | **Agent** | **μg/plate** | **Agent** | **μg/plate** |
| *S. typhimurium* | TA98 | 2-Nitrofluorene | 1.0 | 2-Aminoanthracene | 0.5 |
|  | TA100 | Sodium Azide | 10.0 | 2-Aminoanthracene | 1.0 |
|  | TA1535 | Sodium Azide | 0.5 | 2-Aminoanthracene | 2.0 |
|  | TA1537 | 9-Aminoacridine | 80.0 | 2-Aminoanthracene | 3.0 |
| *E. coli* | WP2 *uvrA* | 4-Nitroquinoline 1-Oxide | 10.0 | 2-Aminoanthracene | 20.0 |

**Table S6. TK parameters of 28-day repeat intravenous dose toxicity study in Sprague Dawley rats (Table S1).***

| **Day** | **Group** | **C_max_ (μg/ml)** | **t_1/2_ (h)** |
| --- | --- | --- | --- |
| Day 1 | Low-Dose | 61 | 0.30 |
|  | Mid-Dose | 96 | 0.31 |
|  | High-Dose | 168 | 0.24 |
| Day 28 | Low-Dose | 117 | 0.33 |
|  | Mid-Dose | 169 | 0.33 |
|  | High-Dose | 196 | 0.60 |

* Mean values pooled across sex.

C_max_: The maximum plasma concentration.

t_1/2_: Half-life.

**Table S7. TK parameters of the 5-Day repeat subcutaneous dose toxicity study in Göttingen minipigs (Table S2).***

| **Day** | **Group** | **C_max_ (μg/ml)** | **t_1/2_ (h)** |
| --- | --- | --- | --- |
| Day 1 | Low-Dose | 13 | 1.8 |
|  | Mid-Dose | 86 | 2.6 |
|  | High-Dose | 147 | 2.1 |
| Day 5 | Low-Dose | 8 | 2.3 |
|  | Mid-Dose | 46 | 3.6 |
|  | High-Dose | 64 | 3.0 |

* Mean values pooled across sex.

C_max_: The maximum serum concentration.

t_1/2_: Half-life.

**Table S8. TK parameters of the 3-Day repeat intradermal dose toxicity study in wounded Göttingen pigs (Table S3).***

| **Group** | **C_max_ (μg/ml)** | **t_1/2_ (h)** |
| --- | --- | --- |
| Low-Dose | 1.5 | 1.3 |
| High-Dose | 3.7 | 1.1 |

* Mean values pooled across sex.

C_max_: The maximum serum concentration.

t_1/2_: Half-life.

**Table S9. Skin irritation assay. EpiDerm^TM^ reconstructed human epidermis model.**

| **Sample** | **Relative viability (%)*** | **Classification** |
| --- | --- | --- |
| Negative control | 100 | Non-irritant |
| Positive control | 2 | Irritant |
| SLI-F06 | 82 | Non-irritant |

* Data were extracted from the report provided by the CRO and presented by mean. N = 3.

**Table S10. Evaluation of the respiratory function of SLI-F06 in rats (Table S4).***

| **Group** | **Time (min)** | **Respiratory rate (breaths/min)** | **Tidal volume (ml)** | **Minute volume (ml)** |
| --- | --- | --- | --- | --- |
| Formulation buffer control | 0 | 208.95 (0) ± 13.06 | 0.88 (0) ± 0.06 | 173.8 (0) ± 3.06 |
|  | 15 | 218.65 (5) ± 15.62 | 0.78 (-11) ± 0.05 | 160.98 (-7) ± 9.08 |
|  | 60 | 225.17 (8) ± 21.25 | 0.75 (-15) ± 0.06 | 157.44 (-10) ± 6.74 |
|  | 120 | 231.73 (11) ± 22.56 | 0.81 (-8) ± 0.08 | 172.06 (-1) ± 10.93 |
|  | 240 | 181.43 (-13) ± 10.83 | 1.01 (15) ± 0.05 | 172.65 (-1) ± 8.19 |
| Low-dose | 0 | 252.01 (0) ± 7.54 | 0.76 (0) ± 0.03 | 183.00 (0) ± 4.31 |
|  | 15 | 249.60 (-1) ± 21.59 | 0.68 (-11) 0.05 | 158.73 (-13) ± 8.03 |
|  | 60 | 217.36 (-14) ± 25.88 | 0.80 (6) ± 0.05 | 163.30 (-11) ± 12.16 |
|  | 120 | 212.83 (-16) ± 16.17 | 0.76 (1) ± 0.07 | 152.82 (-16) ± 15.00 |
|  | 240 | 207.23 (-18) ± 23.35 | 0.79 (4) ± 0.06^&^ | 153.23 (-16) ± 10.56 |
| Mid-dose | 0 | 225.74 (0) ± 18.73 | 0.74 (0) ± 0.03 | 160.37 (0) ± 10.46 |
|  | 15 | 195.37 (-13) ± 16.24 | 0.80 (8) ± 0.05 | 148.06 (-8) ± 8.08 |
|  | 60 | 203.43 (-10) ± 16.10 | 0.70 (-6) ± 0.05 | 137.10 (-15) ± 11.89 |
|  | 120 | 191.89 (-15) ± 15.39 | 0.72 (-3) ± 0.05 | 131.94 (-18) ± 9.43^&^ |
|  | 240 | 180.66 (-20) ± 16.61 | 0.78 (5) ± 0.06^&^ | 137.59 (-14) ± 11.79 |
| High-dose | 0 | 255.03 (0) ± 19.01 | 0.72 (0) ± 0.05 | 175.90 (0) ± 7.43 |
|  | 15 | 208.05 (-18) ± 16.28 | 0.80 (11) ± 0.07 | 157.63 (-10) ± 5.19 |
|  | 60 | 170.51 (-33) ± 10.45^#^ | 0.82 (13) ± 0.05 | 136.43 (-22) ± 11.39^#^ |
|  | 120 | 190.68 (-25) ± 8.92^#^ | 0.77 (6) ± 0.06 | 140.72 (-20) ± 11.62^#^ |
|  | 240 | 158.78 (-38) ± 15.37^#^ | 0.88 (21) ± 0.07 | 131.84 (-25) ± 11.90^#,&^ |

* Data were extracted from the report provided by the CRO and presented by mean (%Δ) ± standard error of mean. N = 6.

^#^ *P* < 0.05 compared to time 0.

^&^ *P* < 0.05 compared to formulation buffer control.

**Supplementary Figures**


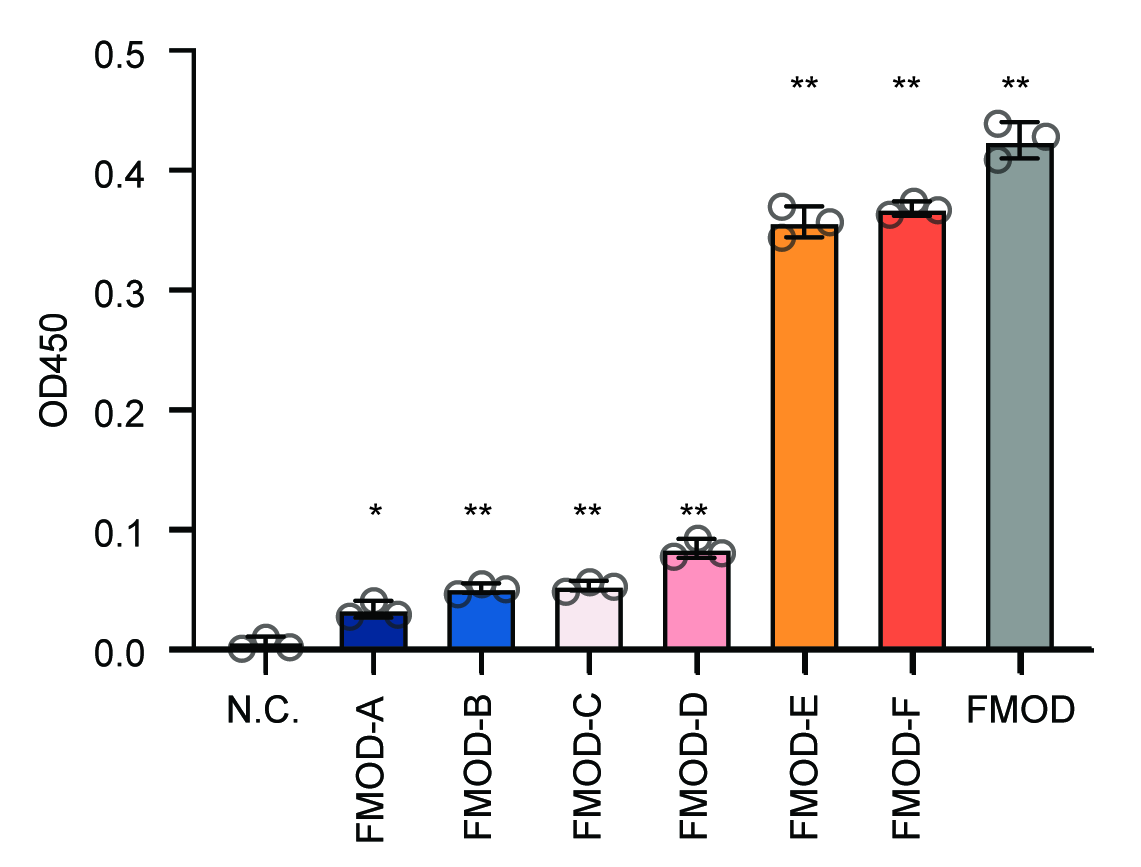


**Figure S1. Initial screening of putative TGFβ1-binding regions of FMOD.**

Based on the FMOD protein structural analysis, various FMOD fragments (only six are shown here) were expressed by the Champion^TM^ pET SUMO Expression System. The expression product of the control plasmid pET_SUMO/CAT was used as the negative control (N.C.), while recombinant SUMO-fused FMOD whole protein served as the positive control. The enzyme-linked immunosorbent assay results suggested that two FMOD regions, E and F, exhibited significant capability binding to TGFβ1. N = 3; *, *P* < 0.05; **, *P* < 0.005, respectively, compared with the negative control.

**
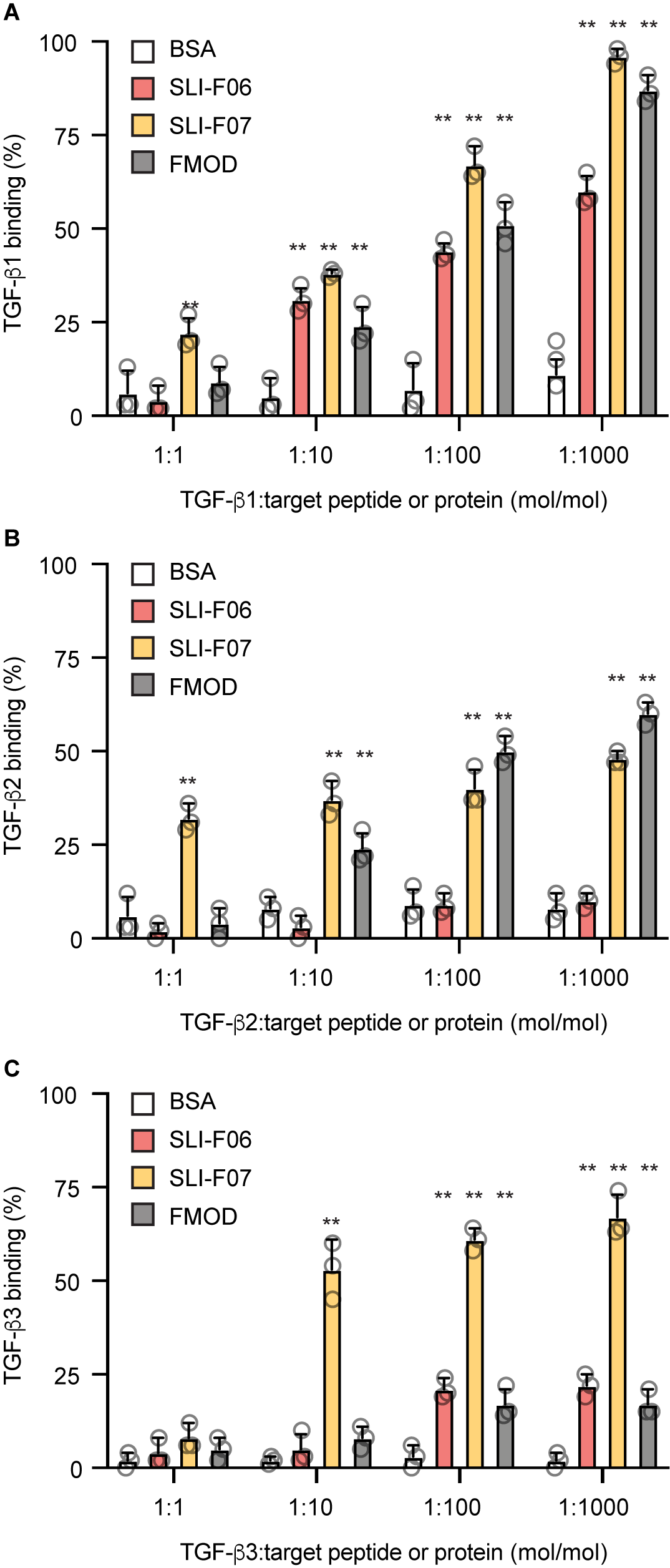
**

**Figure S2. TGFβ-binding activities of synthesized FMOD-derived peptides.**

Biotinylated proteins and peptides were bound to Pierce^TM^ Monomeric Avidin UltraLink^TM^ Resin, followed by incubation with TGFβ1 (**A**), TGFβ2 (**B**), and TGFβ3 (**C**), respectively. Non-bound TGFβs were then collected, sterilized, and quantified by relative growth inhibition of Mv1Lu cells. Data is presented as the percentage of the initially used TGFβs. N = 3; **, *P* < 0.005, compared with the negative control, biotinylated bovine serum albumin (BSA).


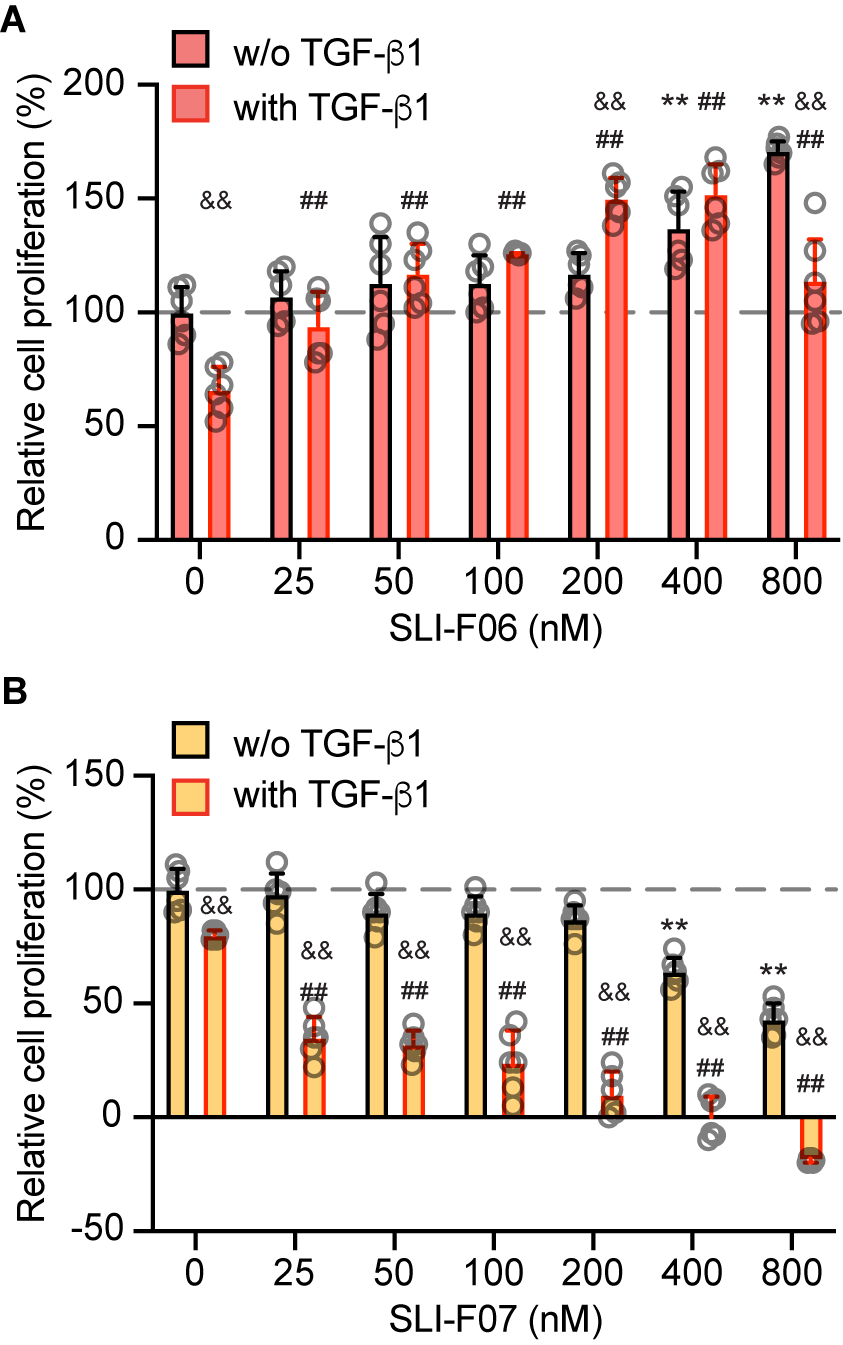


**Figure S3. Different effects of synthesized FMOD-derived peptides on adult RDF proliferation.**

SLI-F06 (**A**) and SLI-F07 (**B**) alone did not significantly affect adult RDF proliferation when applied at concentrations below 200 nM. At higher concentrations (400 nM and 800 nM), SLI-F06 application resulted in increased RDF proliferation, while SLI-F07 led to reduced proliferation. Although TGFβ1 alone inhibited RDF proliferation, SLI-F06 significantly stimulated RDF proliferation in the presence of TGFβ1 (**A**). On the other hand, SLI-F07 markedly enhanced the inhibitory effect of TGFβ1 (**B**). Data were normalized to untreated RDFs (dashed lines). N = 6; *, *P* < 0.05; **, *P* < 0.005, respectively, compared with the vehicle buffer control; ^##^, *P* < 0.005, compared with the group treated with TGFβ1 alone; and ^&&^, *P* < 0.005, comparison between the without and with TGFβ1 groups at the same SLI-F06 (**A**) or SLI-F07 (**B**) concentration.

**
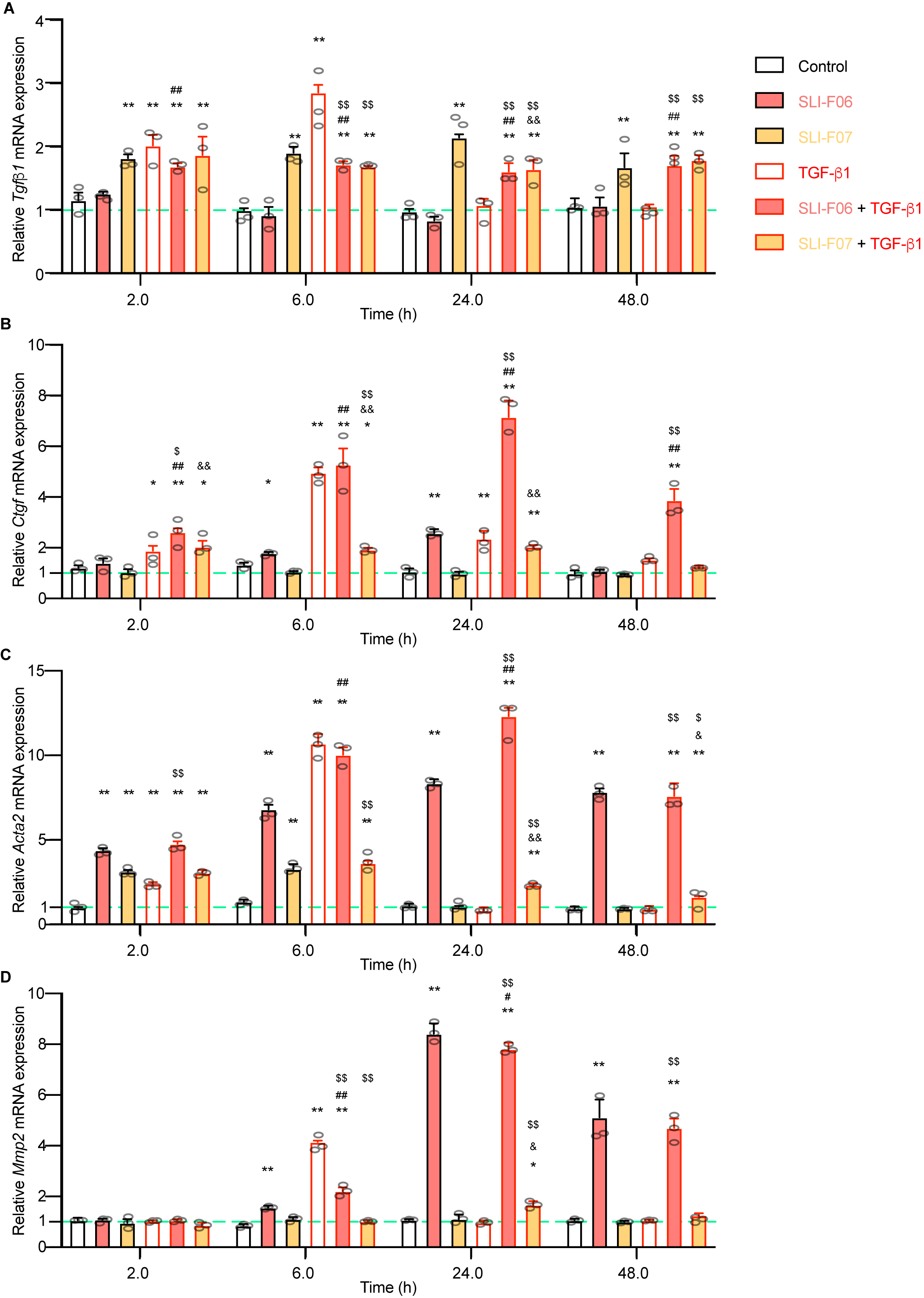
**

**
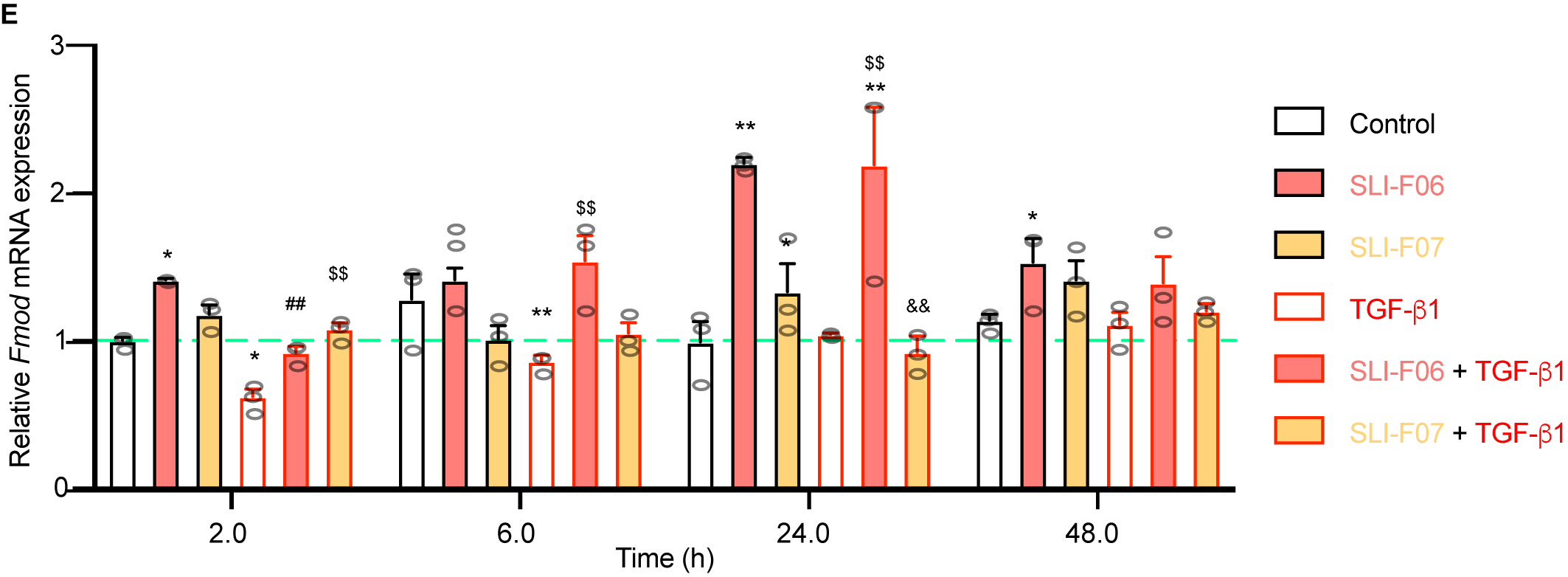
**

**Figure S4. Gene expression of adult RDFs responding to the SLI-F06 and SLI-F07 treatment.**

Expression of *Tgfβ1* (**A**), *Ctgf* (**B**), *Acta2* (**C**), *Mmp2* (**D**), and *Fmod* (**E**) was normalized to untreated RDFs at time 0 (dashed lines). N = 3; *, *P* < 0.05; **, *P* < 0.005, respectively, compared with the vehicle buffer control; ^##^, *P* < 0.005, compared with the group treated with SLI-F06 alone; ^&^, *P* < 0.05; ^&&^, *P* < 0.005, compared with the group treated with SLI-F07 alone; and ^$^, *P* < 0.05; ^$$^, *P* < 0.005, compared with the group treated with TGFβ1 alone.

**
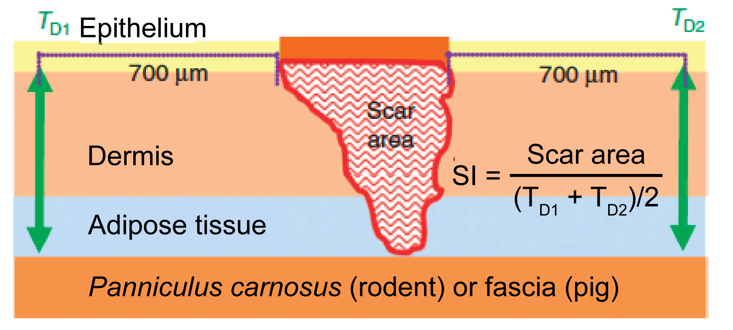
**

**Figure S5. Schematic illustration of scar index (SI) measurement.**

T_D_: the distance between the epidermal-dermal junction down to the *panniculus carnosus* (rodent models) or fascia (pig model). Adopted from our previous publication: Delayed wound closure in fibromodulin-deficient mice is associated with increased TGF-β3 signaling. *J Invest Dermatol* 131 (3): 769-78. https://doi.org/10.1038/jid.2010.381.


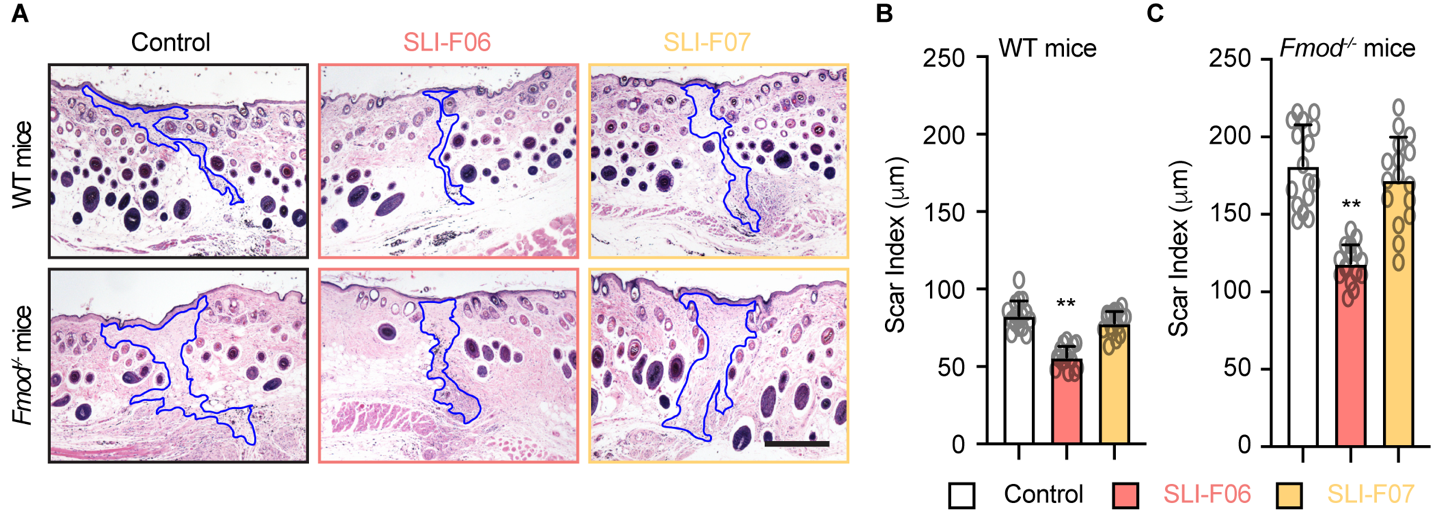


**Figure S6. Different effects of synthesized FMOD-derived peptides in adult mouse wound models.**

Hematoxylin and eosin (H & E) staining was used to elucidate adult wildtype (WT) and *Fmod^-/-^* mouse wounds at day 14 post-injury with the treatment of 2 mg/ml SLI-F06 or SLI-F07 and those who received the vehicle buffer control. Blue lines outline scars (**A**). Scar Index quantified scar size in wildtype (**B**) and *Fmod^-/-^* mice (**C**). Scale bar = 200 μm. N = 16; **, *P* < 0.005, compared with the vehicle buffer control.


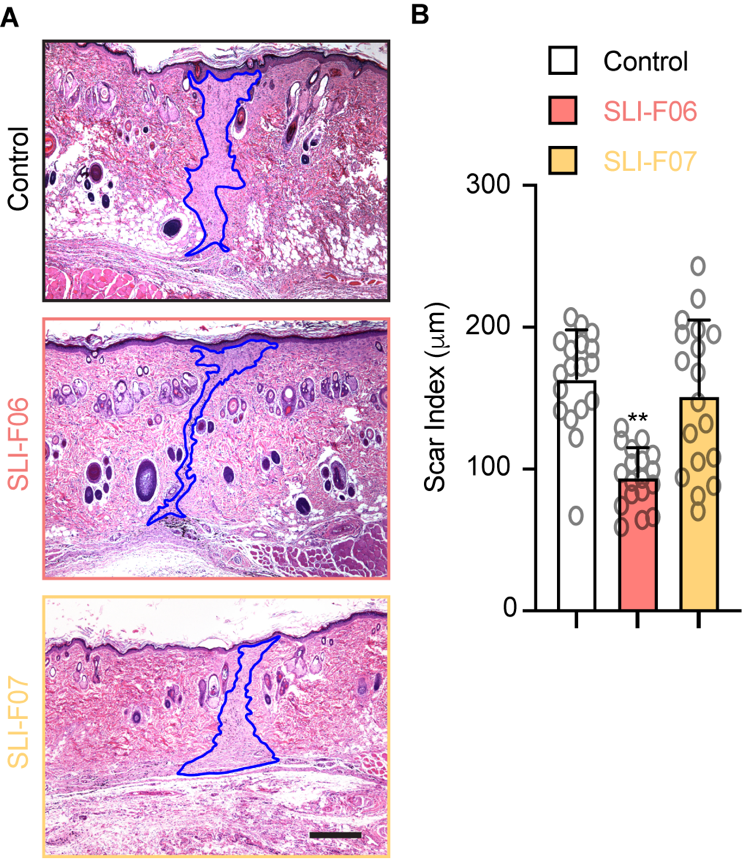


**Figure S7. Different effects of synthesized FMOD-derived peptides in adult rat wound models.**

H & E staining was used to elucidate adult rat wounds at day 14 post-injury treated with vehicle buffer control, 2 mg/ml SLI-F06 or SLI-F07. Blue lines outline scars (**A**). Scar Index quantified scar size (**B**). Scale bar = 200 μm. N = 18; **, *P* < 0.005, compared with the vehicle buffer control.


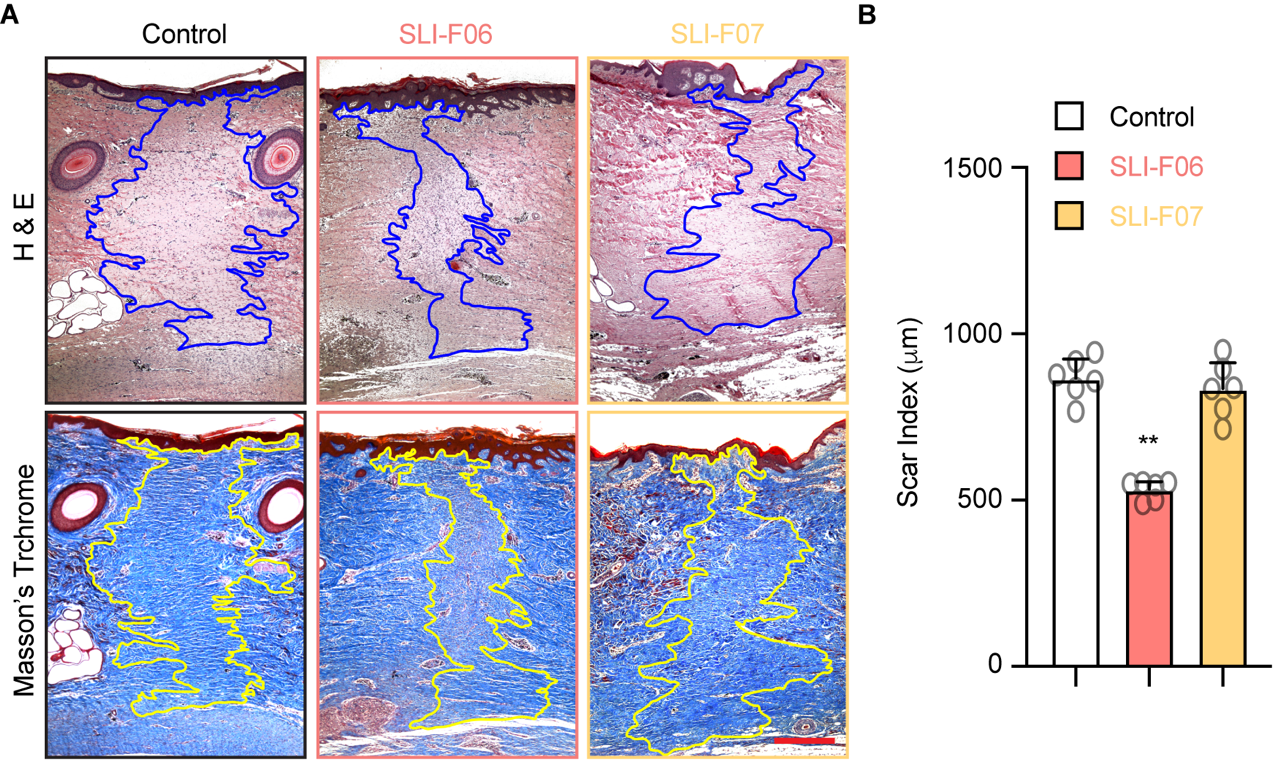


**Figure S8. Different effects of synthesized FMOD-derived peptides in adult Yorkshire pig wounds.**

H & E staining and Masson’s Trichrome staining were used to elucidate the 0.5 cm-width x 1.5 cm-length wounds in adult Yorkshire pigs at week 8 post-injury with the treatment of 2 mg/ml SLI-F06 or SLI-F07 and those who received the vehicle buffer control. Blue (on H & E staining) and yellow (on Masson’s Trichrome staining) lines outline scars (**A**). Scar Index quantified scar size (**B**). Scale bar = 500 μm. N = 6; **, *P* < 0.005, compared with the vehicle buffer control.


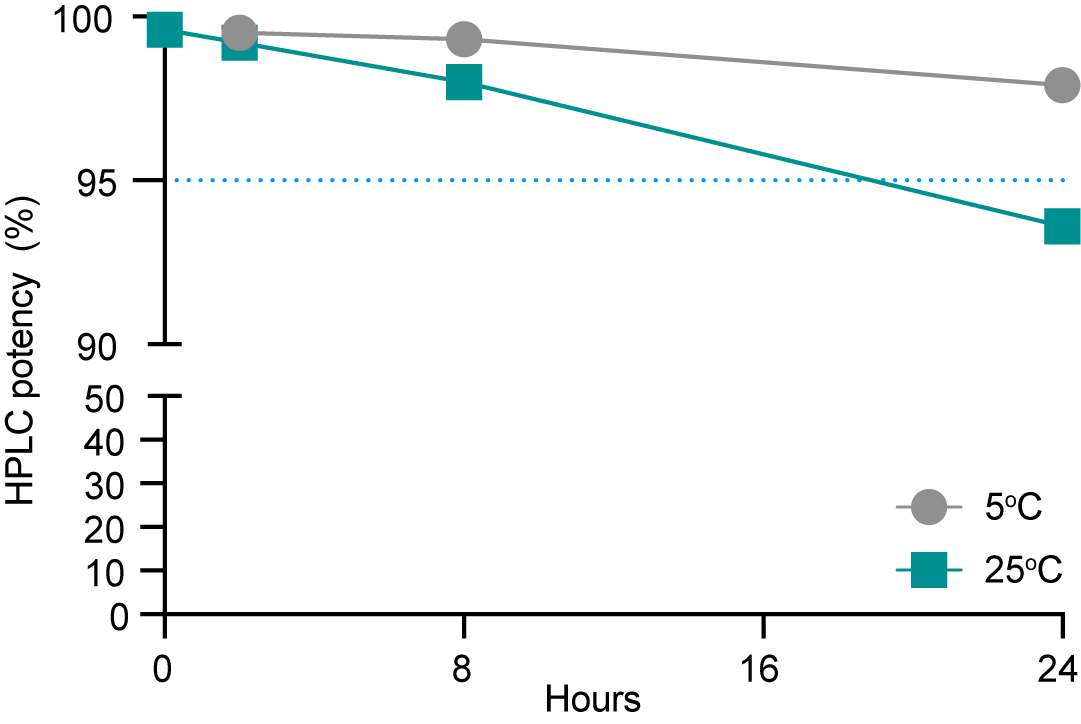


**Figure S9. Stability of SLI-F06 in the formulation buffer.**

HPLC was used to determine the stability of SLI-F06 in the formulation buffer at 5^o^C and 25^o^C. 25 mg/ml SLI-F06 in 50 ml formulation buffer was used for the testing. The testing was conducted by a CRO, pyramid Laboratories Inc.

**References:**

1. Zheng Z, Nguyen C, Zhang X, et al. Delayed wound closure in fibromodulin-deficient mice is associated with increased TGF-beta3 signaling. *J Invest Dermatol*. Mar 2011;131(3):769-78.

2. Li CS, Yang P, Ting K, et al. Fibromodulin reprogrammed cells: A novel cell source for bone regeneration. *Biomaterials*. Jan 7 2016;83:194-206.

3. Freshney RI. *Culture of Animal Cells: A Manual of Basic Technique*. 2nd ed. A. R. Liss; 1987.

4. Zheng Z, James AW, Li C, et al. Fibromodulin reduces scar formation in adult cutaneous wounds by eliciting a fetal-like phenotype. Article. *Signal Transduction And Targeted Therapy*. 10/13/online 2017;2:17050.

5. Zheng Z, Li C, Ha P, et al. *CDKN2B* upregulation prevents teratoma formation in multipotent fibromodulin reprogrammed cells. *Journal of Clinical Investigation*. 2019;129(8):3236-3251.

6. Zheng Z, Lee KS, Zhang X, et al. Fibromodulin-deficiency alters temporospatial expression patterns of transforming growth factor-beta ligands and receptors during adult mouse skin wound healing. *PloS one*. 2014;9(6):e90817.

7. Khorasani H, Zheng Z, Nguyen C, et al. A quantitative approach to scar analysis. *Am J Pathol*. 2011;178:621-628.

8. Jiang W, Ting K, Lee S, et al. Fibromodulin reduces scar size and increases scar tensile strength in normal and excessive-mechanical-loading porcine cutaneous wounds. *Journal of cellular and molecular medicine*. 2018;22(4):2510-2513.

9. Duncan JA, Bond JS, Mason T, et al. Visual analogue scale scoring and ranking: a suitable and sensitive method for assessing scar quality? Randomized Controlled Trial. *Plast Reconstr Surg*. Sep 15 2006;118(4):909-18.
